# Reactive Oxygen Species (ROS)-Sensitive Prodrugs of the Tyrosine Kinase Inhibitor Crizotinib

**DOI:** 10.3390/molecules25051149

**Published:** 2020-03-04

**Authors:** Bjoern Bielec, Isabella Poetsch, Esra Ahmed, Petra Heffeter, Bernhard K. Keppler, Christian R. Kowol

**Affiliations:** 1 Institute of Inorganic Chemistry, Faculty of Chemistry, University of Vienna, Waehringer Strasse 42, 1090 Vienna, Austria; 2 Institute of Cancer Research, Medical University of Vienna, Borschkegasse 8a, 1090 Vienna, Austria; 3 Research Cluster ‘‘Translational Cancer Therapy Research’’, 1090 Vienna, Austria

**Keywords:** anticancer, tyrosine kinase inhibitors, crizotinib, prodrugs, boronic acid

## Abstract

Tyrosine kinase inhibitors revolutionized cancer therapy but still evoke strong adverse effects that can dramatically reduce patients’ quality of life. One possibility to enhance drug safety is the exploitation of prodrug strategies to selectively activate a drug inside the tumor tissue. In this study, we designed a prodrug strategy for the approved c-MET, ALK, and ROS1 tyrosine kinase inhibitor crizotinib. Therefore, a boronic-acid trigger moiety was attached to the 2-aminopyridine group of crizotinib, which is a crucial position for target kinase binding. The influence of the modifications on the c-MET- and ALK-binding ability was investigated by docking studies, and the strongly reduced interactions could be confirmed by cell-free kinase inhibition assay. Furthermore, the newly synthesized compounds were tested for their activation behavior with H_2_O_2_ and their stability in cell culture medium and serum. Finally, the biological activity of the prodrugs was investigated in three cancer cell lines and revealed a good correlation between activity and intrinsic H_2_O_2_ levels of the cells for prodrug **A**. Furthermore, the activity of this prodrug was distinctly reduced in a non-malignant, c-MET expressing human lung fibroblast (HLF) cell line.

## 1. Introduction

Tyrosine kinases catalyze the transfer of the γ-phosphate groups of adenosine triphosphate (ATP) between enzymes and play a central role in cell signaling pathways from the cell surface to the nucleus [1]. Therefore, they are involved in cellular processes like differentiation, proliferation, and apoptosis. In cancer cells, these proteins are frequently activated in an aberrant manner that can lead to tumor formation or metastasis [2]. Inhibition of these enzymes is a successful strategy in anticancer therapy and has led to the approval of several different classes of targeted therapeutics [3,4]. Unfortunately, tyrosine kinases are likewise expressed in healthy tissue, and drugging of these targets often results in a broad toxicity profile caused by off-target effects (due to the lack of target specificity), and on-target toxicities (based on target inhibition in healthy tissues) [5].

The class of tyrosine kinase inhibitors (TKIs) consists of small-molecule anticancer therapeutics competing with ATP in the active sites of tyrosine kinases, most frequently in a reversible and competitive manner [3]. High intracellular ATP concentrations of up to 5 mM shift the inhibition potency of TKIs from typical cell-free IC_50_ values in the low nM to the µM range [6]. The efficacy of an inhibitor is determined by its dissociation constant that relies on the chemical structure of the drug and its interaction profile with the active site of the enzyme. A reversible chemical modification of a drug at a crucial binding position may lead to a higher dissociation constant and lower drug potency. This provides an ideal basis for prodrug strategies, where the drug is specifically activated inside the tumor which prevents undesired on- and off-target effects in healthy tissues. While many different prodrug approaches have been reported for chemotherapeutics like antibiotics [7], antimetabolites [8] or alkylating agents [9], so far there are just a few examples known for the class of TKIs [10,11]. 

**Scheme 1:**
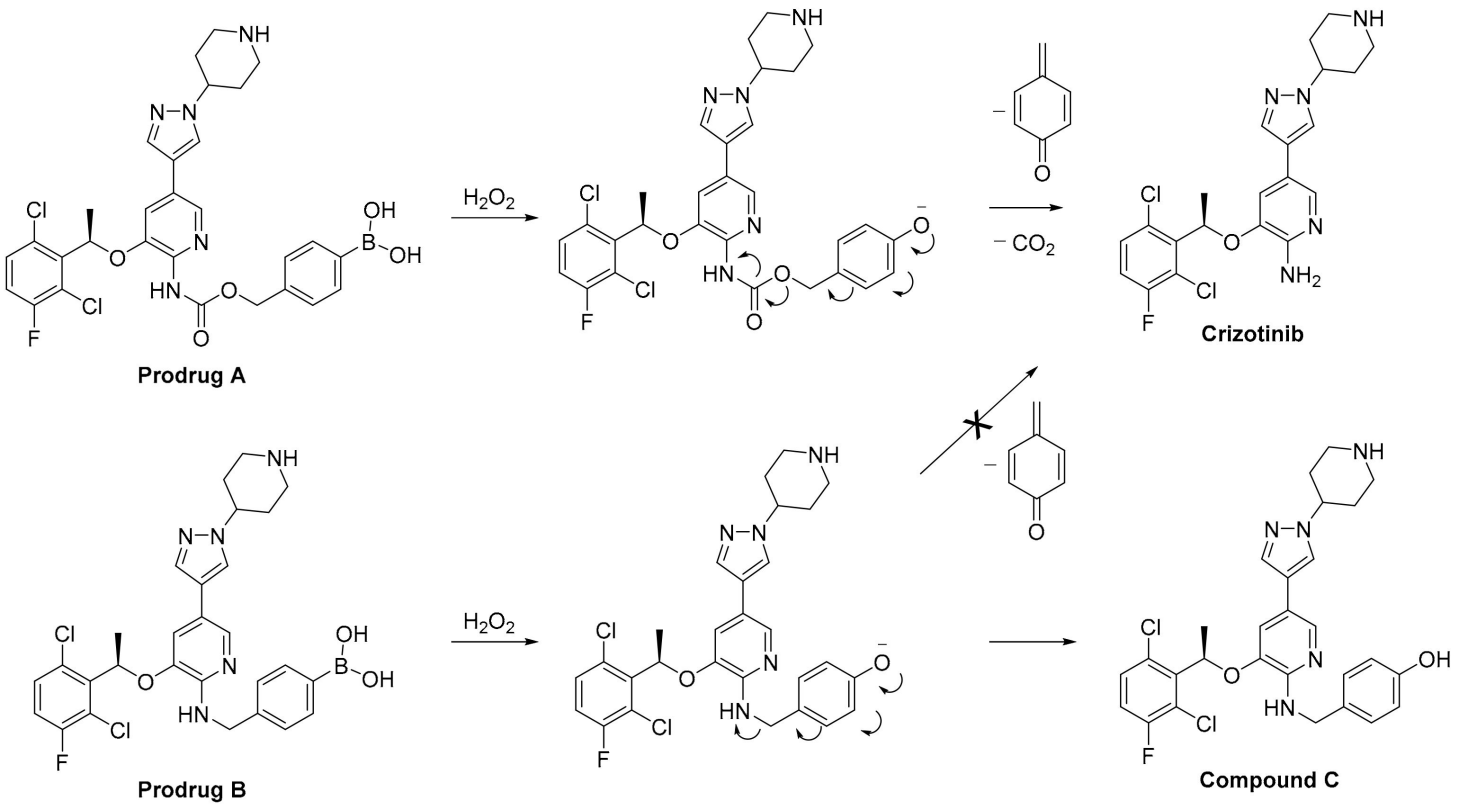
Activation scheme of the boronic acid trigger residue of prodrugs **A** and **B** upon oxidation with H_2_O_2_.

Crizotinib (Xalkori^®^) has initially been developed as a c-MET inhibitor by Pfizer^®^ in 2007 [12]. However, crizotinib was also found to be a potent inhibitor of the Echinoderm Microtubule associated protein-Like 4-Anablastic Lymphoma Kinase (EML4-ALK) fusion protein and the ROS proto-oncogene 1 tyrosine-protein kinase (ROS1) which led to its U.S. Food and Drug Administration (FDA) approval for treatment of ALK-positive non-small cell lung cancer (NSCLC) in 2011 [13] and ROS1-postive NSCLC in 2016 [14]. The most common grade 3/4 side effects include alanine aminotransferase elevation (hepatotoxicity) and neutropenia [15] leading to dose reduction or even discontinuation of treatment [16]. The structure of crizotinib (Scheme 1) comprises a 2-aminopyridine functional moiety that forms crucial bonds with amino acids in the ATP-binding pocket of all three target kinases (i.e., ALK, c-MET, and ROS1) [3] and, thus, can be considered as an ideal moiety for prodrug derivatization.

In the literature, different strategies have been described to activate prodrugs, specifically in the tumor tissue. The most prominent strategies are based on enzymatic [17] (e.g., capecitabine), reductive [18] (e.g., evofosfamide) or oxidative [19] activation. One characteristic difference of cancer cells is an up to ten-fold higher concentration of reactive oxygen species (ROS) like hydrogen peroxide (H_2_O_2_) [20] compared to healthy tissues [21]. As a trigger moiety for ROS-mediated prodrug activation, boronic acids have been used, for example, for anticancer drugs like aminoferrocene [22], antifolates [23] or several alkylating agents [24]. In the case of phenylboronic acid trigger units, upon reaction with H_2_O_2_, the generated boric acid is readily hydrolyzed, and the free drug is released via an electron-migration cascade with simultaneous formation of a quinone methide species from the linker moiety (Scheme 1).

In this work, we describe the design of the first ROS-sensitive prodrugs of crizotinib, where the trigger moiety was introduced via carbamoylation (prodrug **A**) or alkylation (prodrug **B**) of the 2-aminopyridine moiety (Scheme 1). We examined their binding profiles to ALK and c-MET through molecular docking studies, evaluated their activation behavior by incubation with H_2_O_2_, their efficacy against ALK and c-MET in a cell-free protein assay, their stability under physiological conditions, and the biological activity in cell culture.

## 2. Results and Discussion

### 2.1. In Silico Studies

#### 2.1.1. Docking Studies

**Figure 1:**
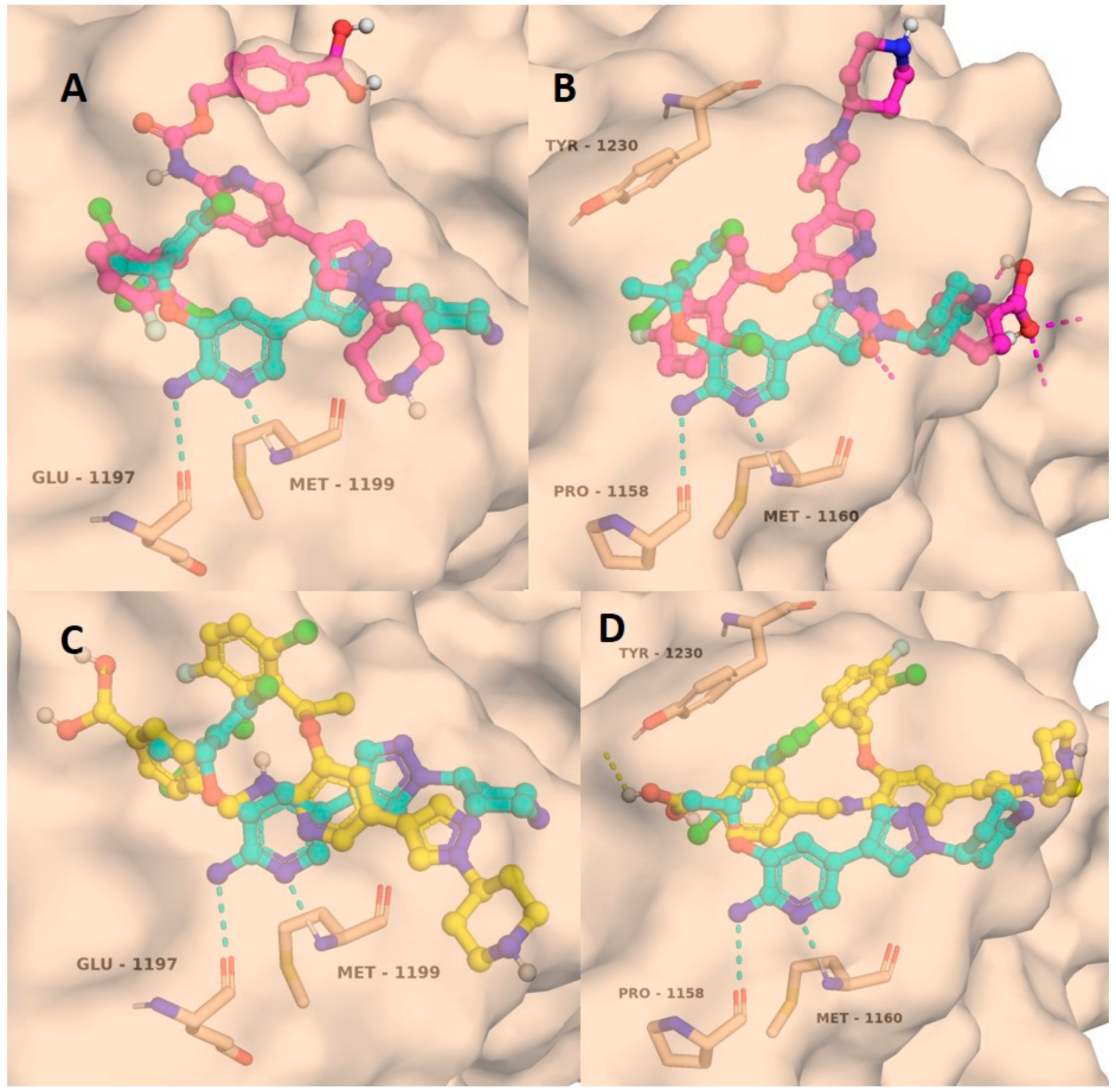
Best docking binding modes of prodrug **A** with ALK (**A**, RCSB protein data bank (PDB) ID: 2XP2) and c-MET (**B**, PDB ID: 2WGJ) as well as prodrug **B** with ALK (**C**) and c-MET (**D**) compared to crizotinib. Crizotinib is shown in cyan, prodrug **A** in magenta, and prodrug **B** in yellow. Hydrogen bonds are shown in dashed lines in the same color as the donor/acceptor compounds.

Reversible tyrosine kinase inhibitors have been designed to exhibit strong intermolecular, non-covalent interactions with their specific targets. Simplified, these interactions can be described by the sum of hydrogen bonds, van-der-Waals, and coulomb interactions [25]. In order to verify the reduced ability to enter and form strong bonds with the ATP-binding pockets of ALK and c-MET, the ligand-binding modes of both prodrugs were compared with crizotinib via molecular docking studies. The results of these studies indicated a complete suppression of the crucial hydrogen bonds of crizotinib’s 2-aminopyridine moiety with Glu–1197 and Met-1199 for both prodrugs in the ALK-binding pocket (Figure 1A,C and Figure S1A). In the case of c–MET, the main interactions formed of the 2-aminopyridine moiety with Pro–1158 and Met–1160 and additionally a crucial π-stacking of crizotinib’s 2,6-dichloro-3-fluorophenyl ring with the Tyr–1230 residue were prohibited (Figure 1B,D and Figure S1B). Notably, the boronic acid residue formed hydrogen bonds with different amino acids in all cases. All interactions of the prodrugs with both tyrosine kinases are visualized in 2D plots in Figure S2.

The docking studies clearly show that the derivatization of the 2-aminopyridine moiety of crizotinib with a phenylboronic acid moiety can be considered as a valuable prodrug strategy to reduce or even suppress the crucial interactions between crizotinib and both tyrosine kinases.

#### 2.1.2. In Silico ADME Prediction

In addition, the “drug-likeness” profiles of both prodrugs were evaluated by in silico adsorption, distribution, metabolization, excretion (ADME) prediction, and compared to crizotinib using the SwissADME web tool [26]. The physicochemical properties of prodrugs **A** and **B** are summarized in Table 1 and visualized in BOILED-Egg plots in Figure S3. Due to the structural modification of crizotinib, for both prodrugs the numbers of H-bond acceptors and H-bond donors increased but were still in agreement with Lipinski’s rule of five. Likewise, the other Lipinski rules were satisfied, except the molecular weight > 500 g/mol. The topological polar surface area (TPSA) is described by the sum of the polar surfaces of all fragments of a molecule and is used to predict intestinal absorption, cell monolayer and blood–brain barrier penetration, as well as oral bioavailability [27]. In the case of both prodrugs, the TPSA remained ≤ 140 Å^2^ and would be sufficient for cellular uptake. Furthermore, both prodrugs showed similar lipophilicities in the range of crizotinib, but the calculated aqueous solubilities were lower than for the parental drug. Notably, the high TPSA of prodrug **A** led to the prediction of a low gastrointestinal (GI) absorption as visualized in the BOILED-Egg plots in Figure S3. However, this value was located at the borderline to high absorption ratios and also some approved orally applied drugs like lapatinib are predicted to possess low GI absorption [26]. Remarkably, in contrast to prodrug **A**, crizotinib and prodrug **B** were predicted as P-glycoprotein substrates and can therefore be actively transported out of cells. 

### 2.2. Synthesis

**Scheme 2:**
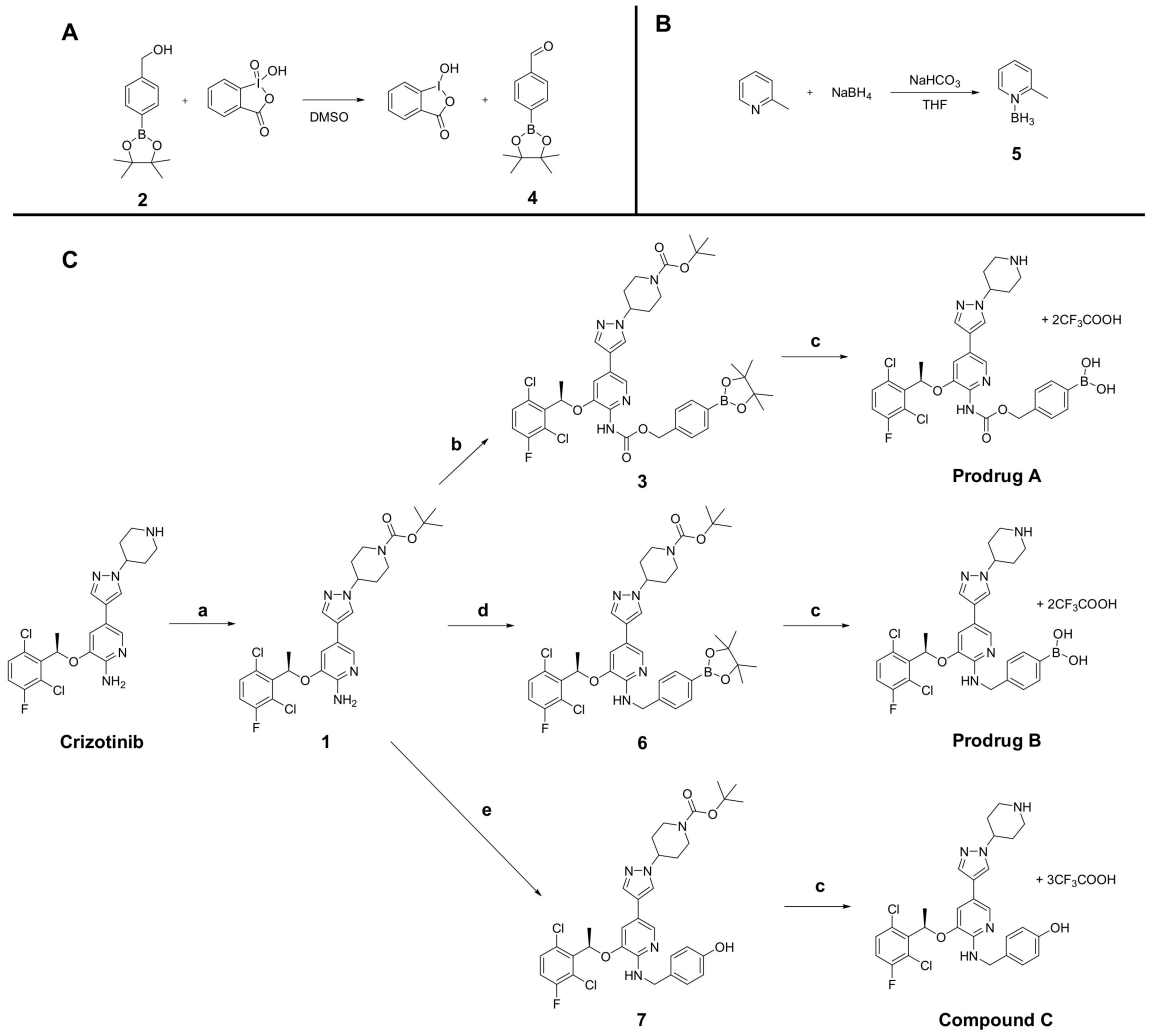
(**A**) Synthesis of aldehyde **4**. (**B**) Synthesis of picolin–borane **5.** (**C**) Synthesis of the crizotinib prodrugs **A** and **B**. **a**) Di-tert-butyl dicarbonate, THF, 12 h; **b**) **2**, carbon monoxide, Pd(OAc)_2_, Cu(OAc)_2_ H_2_O, KI, DMSO, ACN; **c**) TFA, DCM; **d**) **4**, **5**, AcOH/MeOH; **e**) 4-hydroxybenzaldehyde, **5**, AcOH/MeOH.

In order to selectively modify the 2-aminopyridine functionality of crizotinib, the piperidinic nitrogen was protected (compound **1**, Scheme 2) using a tert-butyloxycarbonyl (boc) protecting strategy to generate a water-soluble salt species after deprotection in the final step. The ROS-sensitive trigger moiety was then introduced via a carbamate (prodrug **A**), utilizing a carbamoylation method or via alkylation (prodrug **B**), exploiting a reductive amination approach. For the synthesis of prodrug **A**, the pinacol boronic acid alcohol **2** (Scheme 2C) was reacted with boc-protected crizotinib **1** in presence of Cu(OAc)_2_, Pd(OAc)_2_, and potassium iodide in a mixture of acetonitrile and dimethyl sulfoxide (DMSO), forming the carbamate intermediate **3**. Deprotection of the boc-moiety was performed using a mixture of trifluoroacetic acid (TFA) in dichloromethane to yield the corresponding TFA salt of prodrug **A**. However, standard procedures described in the literature to couple compound **2** via transformation into a chloroformate did not yield the desired product **3** [23]. In the case of the alkylated prodrug **B**, alcohol **2** was oxidized to the corresponding aldehyde **4** using 2-iodobenzoic acid (IBX) in DMSO (Scheme 2A). The resulting aldehyde **4** was further reacted with **1** in the presence of 2-picoline borane (**5**, Scheme 2B) as a reducing agent, yielding the desired product **6**. Interestingly, other standard reducing agents for reductive amination approaches (e.g., sodium triacetoxyborohydride) did not yield the desired product. The alkylated crizotinib intermediate **6** was further deprotected with TFA leading to the respective salt species of prodrug **B**.

In both cases, the deprotected products were purified by preparative HPLC. During purification, the pinacol ester of the prodrugs was hydrolyzed in the acidic environment of the eluent yielding the free boronic acid species. Notably, prodrug **A** was initially also purified via column chromatography leading to the intact pinacol-protected prodrug. However, stability studies of this compound revealed fast hydrolysis of the pinacol ester at physiological pH (phosphate buffer at pH 7.4) already after 1 h. This is a very interesting observation, as most publications in the literature focus on the synthesis of the pinacol ester boronic acid derivatives [28]. Therefore, the unprotected boronic acids can be considered as the active pharmaceutical components. Consequently, we focused on the direct synthesis of the boronic acids via in situ deprotection during preparative HPLC purification. Attempts to synthesize the boronic acid prodrugs without initial pinacol protection failed. All new compounds were investigated via NMR, UV/VIS-HPLC measurements, and high-resolution mass spectrometry. Purity was confirmed by elemental analysis.

Next, the stability of both prodrugs was tested in 50 mM phosphate buffer at pH 7.4 (PB7.4) at 37 °C for 72 h to evaluate their structural integrity over time. No significant changes could be observed over the whole period indicating high aqueous stability at physiological conditions (Figure S4).

### 2.3. Kinase Screening

**Figure 2:**
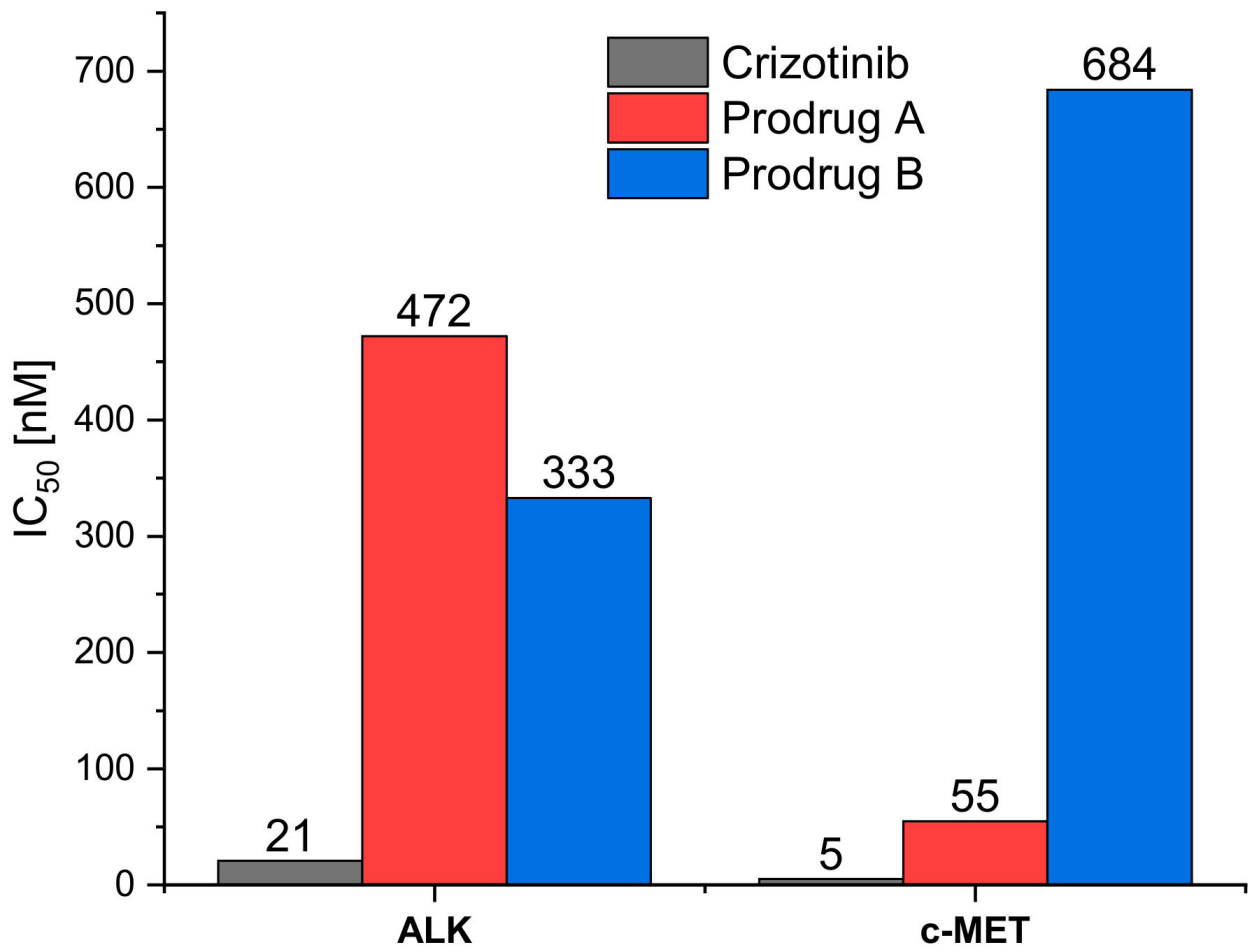
Diagram of the kinase inhibition potency of crizotinib compared to prodrugs **A** (red) and **B** (blue) against ALK and c-MET.

In order to evaluate the prodrug potential of both new compounds, they were tested in a cell-free kinase inhibition assay in the presence of a ten-fold excess of ATP compared to crizotinib (Figure 2, Table S2, and Figure S5). Crizotinib showed IC_50_ values of 21 and 5 nM against ALK and c-MET, respectively, which aligns with the data from literature [12]. In the case of both prodrugs, the inhibition potential was dramatically reduced against both kinases. The activity of prodrug **A** against c-MET showed a ten-fold decrease, while the inhibition of ALK was suppressed by a factor of 22. Prodrug **B** showed even lower activity compared to crizotinib with a factor of 16 in the case of ALK and factor of 137 against c-MET. Hence, the data of the kinase inhibition assay confirmed the results of the docking studies and indicated encouraging properties for both prodrug compounds. 

### 2.4. Activation Assay

**Figure 3:**
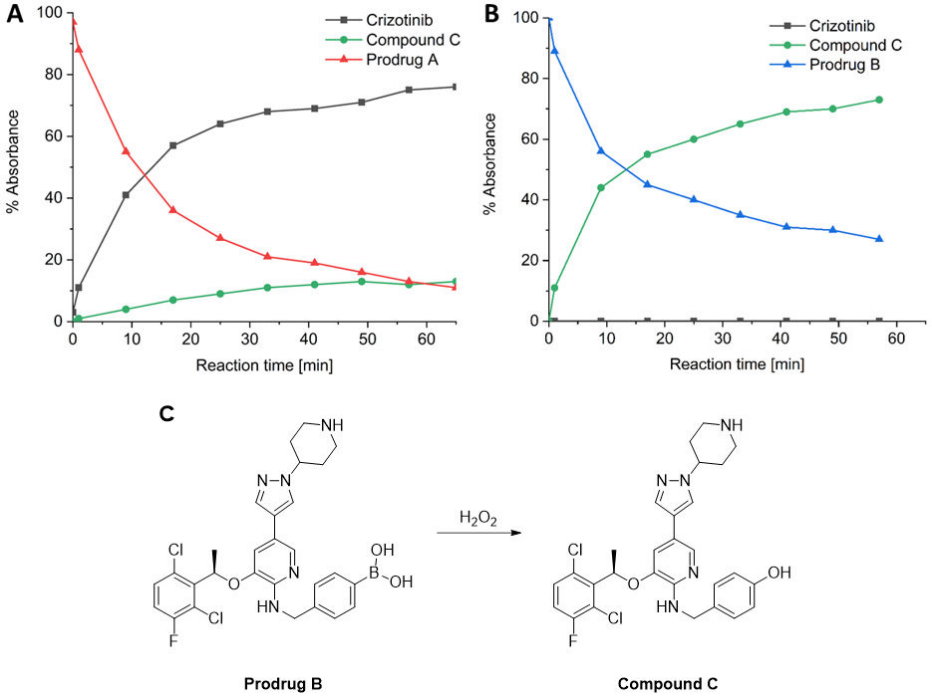
(**A**,**B**) Activation of prodrug **A** and prodrug **B** in phosphate buffer (50 mM) at pH 7.4 and 37 °C for 1 h in the presence of ten equivalents of H_2_O_2_. In the case of prodrug **A**, after 10 min, ~50% of crizotinib was released. (**C**) In the case of prodrug **B**, the activation led to the formation of compound **C** rather than the release of crizotinib.

Inside the tumor tissue, both prodrugs are considered to be activated by H_2_O_2_ to release crizotinib. Therefore, the activation process was verified by incubation with ten equivalents of H_2_O_2_ in phosphate buffer (50 mM, pH 7.4) at 37 °C. As visualized in Figure 3A, the activation of prodrug **A** led to the release of ~50% free crizotinib after 10 min and ~75% after 1 h with only minor formation of a side product (Figure 3C, Figure S6). The HPLC-MS measurements revealed that this side product (*m*/*z*: 556) was most likely a reaction of released crizotinib with the formed quinone methide species. In a complex biological environment, this quinone methide usually reacts with other nucleophiles; consequently, this side reaction can be neglected. Prodrug **B** exhibited a very similar activation kinetic (Figure S7); however, the released compound did not fit in the retention time and mass to crizotinib. The HPLC-MS investigations revealed that the activation led to the formation of a hydrolyzed but stable phenol derivative, compound **C**. Notably, compound **C** had the same mass (*m*/*z* 556) as the formed side product during the activation of compound **A**, although the pathway for this formation was different (Figure 3C). These results indicate that the derivatization of crizotinib via alkylation of the 2-aminopyridine moiety was not reversible and consequently not usable as prodrug system. 

### 2.5. Stability Under Biological Conditions

**Figure 4:**
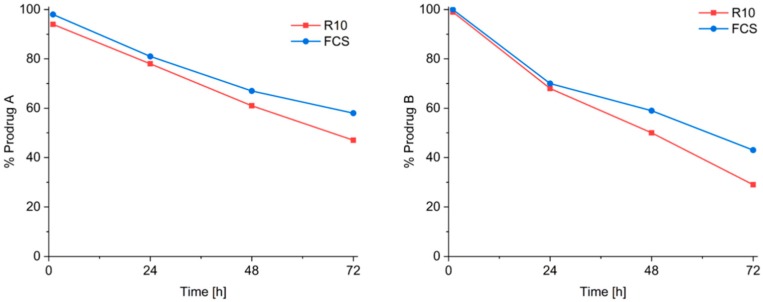
Stability of prodrug **A** (left) and prodrug **B** (right) in RPMI-1640 medium containing 10% FCS (R10, red line) and pure fetal calf serum (FCS, blue line) for up to 72 h.

Next, the prodrugs were incubated in RPMI-1640 medium containing 10% fetal calf serum (R10) and pure fetal calf serum (FCS) for up to 72 h at 37 °C. After precipitation of the macromolecules with acetonitrile, the supernatant was investigated via HPLC-MS measurements (Figure 4). After incubation for 24 h, ~70–80% of the prodrugs were still intact. In case of prodrug **A**, approximately half of the compound was degraded to crizotinib in both R10 and fetal calf serum after 72 h. In case of prodrug **B,** ~30–40% was still intact in R10 medium and in pure FCS after 72 h. As expected from the results of the activation assay, prodrug **B** decomposed to the hydroxybenzyl derivative compound **C** (Figure 3C). As compound **C** was generated during both the activation process and the stability assessment in biological media, we also synthesized and chemically characterized compound **C** for further investigations (see experimental details in Section 4.1).

### 2.6. ROS Production by Cancer Cell Lines

**Figure 5:**
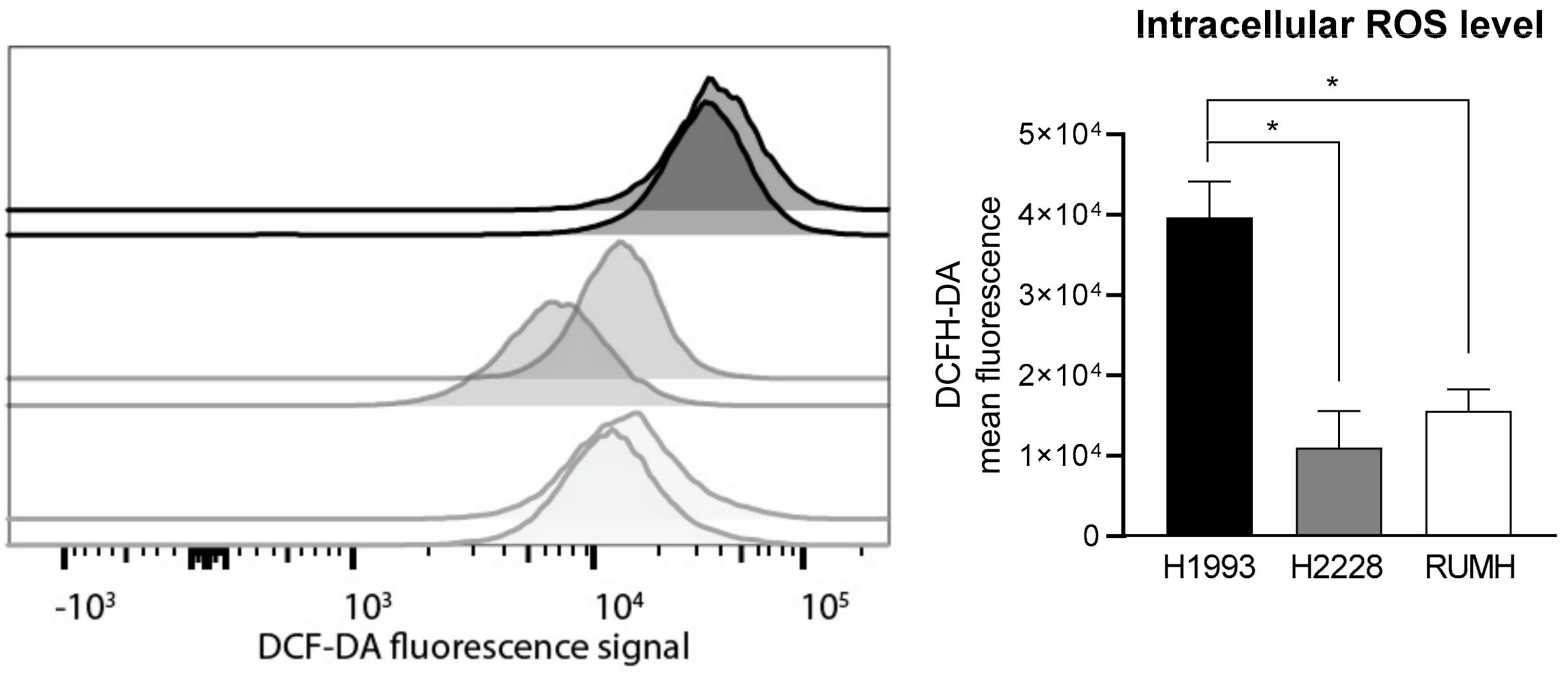
Intracellular ROS levels. Cells were stained with 2′,7′-dichlorofluorescein diacetate (DCFH-DA) to measure basal ROS levels. Samples were analyzed by flow cytometry and fluorescence signals were quantified using FlowJo and Graphpad Prism software. Bars depict mean ± SD. * *p* > 0.05, ordinary one-way ANOVA, multiple comparisons.

To investigate the potential anticancer activity of the prodrugs, three human cancer cell lines were selected that are known for their dependency on c-MET or ALK and, thus, sensitivity to crizotinib treatment: H1993 (c-MET-dependent non-small cell lung cancer), RUMH (c-MET-overexpressing renal cell carcinoma), and H2228 (ALK-dependent non-small cell lung cancer). As the activation of the prodrugs was supposed to be based on intracellular H_2_O_2_ concentrations, as a first step, the ROS levels of the chosen cancer cell models were determined by flow cytometry using 2′,7′-dichlorofluorescein diacetate (DCFH-DA). As shown in Figure 5, H1993 showed the highest basal intracellular ROS levels (4-fold and 2.7-fold higher compared to H2228 and RUMH, respectively).

### 2.7. Cytotoxicity

**Figure 6:**
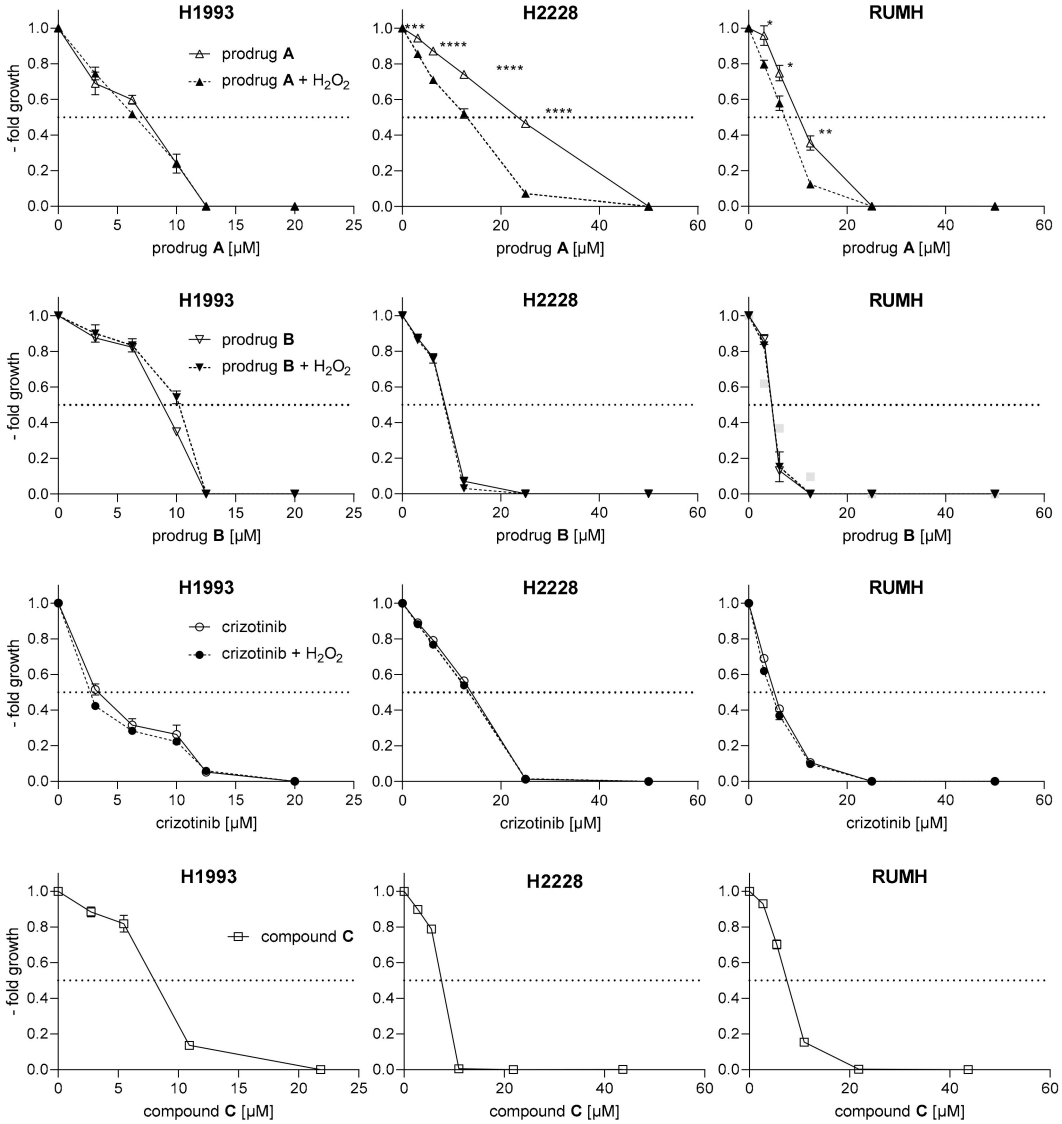
Anticancer activity of drugs −/+ H_2_O_2_ pre-incubation in the three cancer cell lines H1993, H2228, and RUMH. Drug-induced cytotoxicity against the indicated cancer models was measured using MTT assay after 72 h of drug exposure. The values of the samples containing untreated cells were set to 1. Results are given as mean ± SD of triplicates from one representative experiment performed in triplicates. * *p* < 0.05, ** *p* < 0.01, *** *p* < 0.001, **** *p* < 0.0001 (multiple *t*-tests).

Subsequently, these cells were tested for their sensitivity to prodrug **A** and **B** as well as compound **C** and crizotinib by MTT assays after 72 h treatment. In addition, to assess the amount of prodrug activation in these models, the cytotoxicity of the activated drugs after (cell-free) pre-incubation with H_2_O_2_ was tested for comparison. These experiments revealed that only H1993 cells (characterized by the highest intracellular ROS levels) were able to efficiently activate prodrug **A**, indicated by similar IC_50_ values of prodrug **A** with and without prior activation by H_2_O_2_ (Table 2 and Figure 6). In contrast, the activity of pre-activated prodrug **A** was in the range of crizotinib in both of the other tested cell models and distinctly higher than without H_2_O_2_ incubation. In good agreement with the prodrug nature of **A**, the compound displayed distinctly enhanced activity in H2228 and RUMH cells after H_2_O_2_ pre-incubation. Additionally, prodrug **A** was significantly less toxic compared to crizotinib or prodrug **B** in non-malignant HLF cells (up to 2.66-fold). Pre-activation with H_2_O_2_ did not impact on the IC_50_ values of prodrug **B** or crizotinib. Interestingly, although prodrug **B** could not be activated to release crizotinib, the cytotoxicity evaluation revealed comparable IC_50_ values as those of crizotinib in RUMH and H2228 cells. This is in line with the IC_50_ values of the released compound **C** and comparable to prodrug **B** + H_2_O_2_. Additional docking studies with ALK and c-MET (Figure S8, Table S3) provided reasonable explanations for the unexpectedly high activity of compound **C** in cancer cells: In contrast to prodrug **A** and **B**, compound **C** seems to be still able to bind to ALK in a conformation quite similar to crizotinib with a hydrogen bond between the pyridine nitrogen and the ATP-binding site (Met-1199) (Figure S8A). In addition, the phenolic hydroxyl group forms a hydrogen bond with Lys-1150 (Figure S8A). In the case of c-MET, the phenolic hydroxyl group is able to substitute the 2-aminopyridyl moiety and forms a hydrogen bond to the backbone amine of Met-1160 while preserving the crucial π-stacking of the 2,6-dichloro-3-fluorophenyl ring with the Tyr-1230 residue (Figure S8B). Consequently, it seems that the hydroxybenzyl substituent at the 2-aminopyridine moiety is not able to sufficiently suppress binding to the target kinases.

Taken together, these data indicate that also in cell culture, prodrug **A** can only be activated by cancer cells characterized by elevated intracellular ROS levels, whereas prodrug **B** decomposes to compound **C** which is still able to bind to c-MET and ALK and, thus, exhibits IC_50_ values similar to crizotinib.

## 3. Conclusions

In general, derivatization of TKIs into prodrugs is a valuable strategy to improve their tissue selectivity and to reduce on- and/or off-target side effects. In this study, a prodrug strategy based on boronic acids and oxidative cleavage for the TKI crizotinib was designed. Two prodrugs were synthesized via either carbamoylation or alkylation of crizotinib’s 2-aminopyridine moiety. Both drugs were then tested in a cell-free kinase inhibition assay and showed distinctly reduced activity against crizotinib’s targets ALK and c-MET. Furthermore, during incubation of both drugs with H_2_O_2_, prodrug **A** was readily activated within 1 h. Stability measurements of the prodrugs showed good to moderate stability for both prodrugs in FCS for up to 72 h. The MTT assays in three different c-MET-dependent or -overexpressing as well as ALK-dependent cell lines revealed a significant correlation of activity and intracellular H_2_O_2_ levels for prodrug **A**. In addition, the activity of this prodrug was distinctly reduced in the non-malignant, c-MET expressing cell line HLF.

Taken together, the here presented prodrug **A** is a first important step in the development of crizotinib derivatives with tumor-specific activation. The data of the cell-free kinase inhibition assay clearly show that modification of the 2-aminopyridine moiety is a valuable prodrug strategy. A next step could be fine-tuning of the oxidation potential of the boronic acid trigger moiety to generate derivatives with further improved prodrug potential.

## 4. Materials and Methods

### 4.1. Synthesis and Characterization

**Figure 7:**
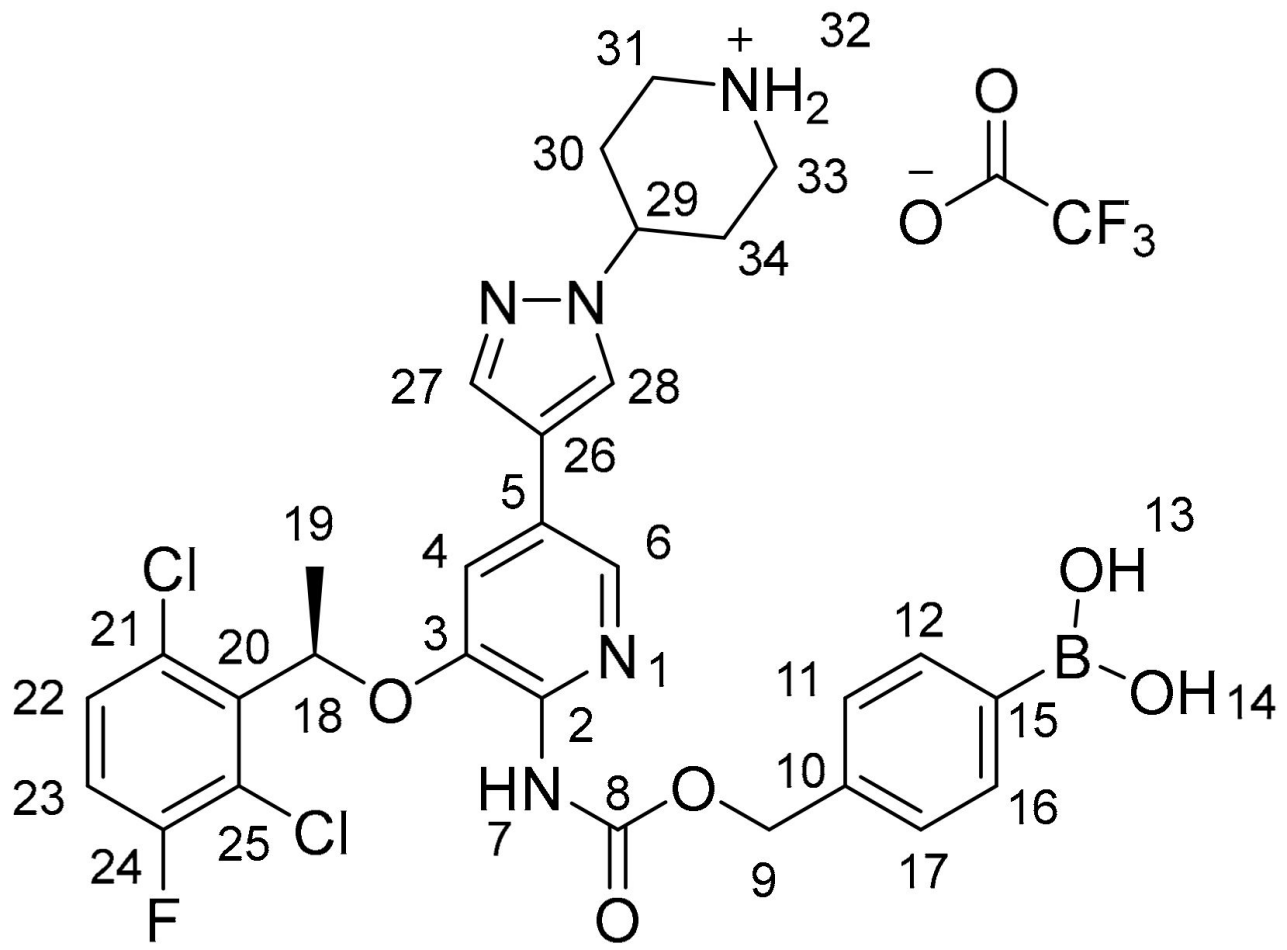
Structure and NMR numbering of prodrug **A**.

**Figure 8:**
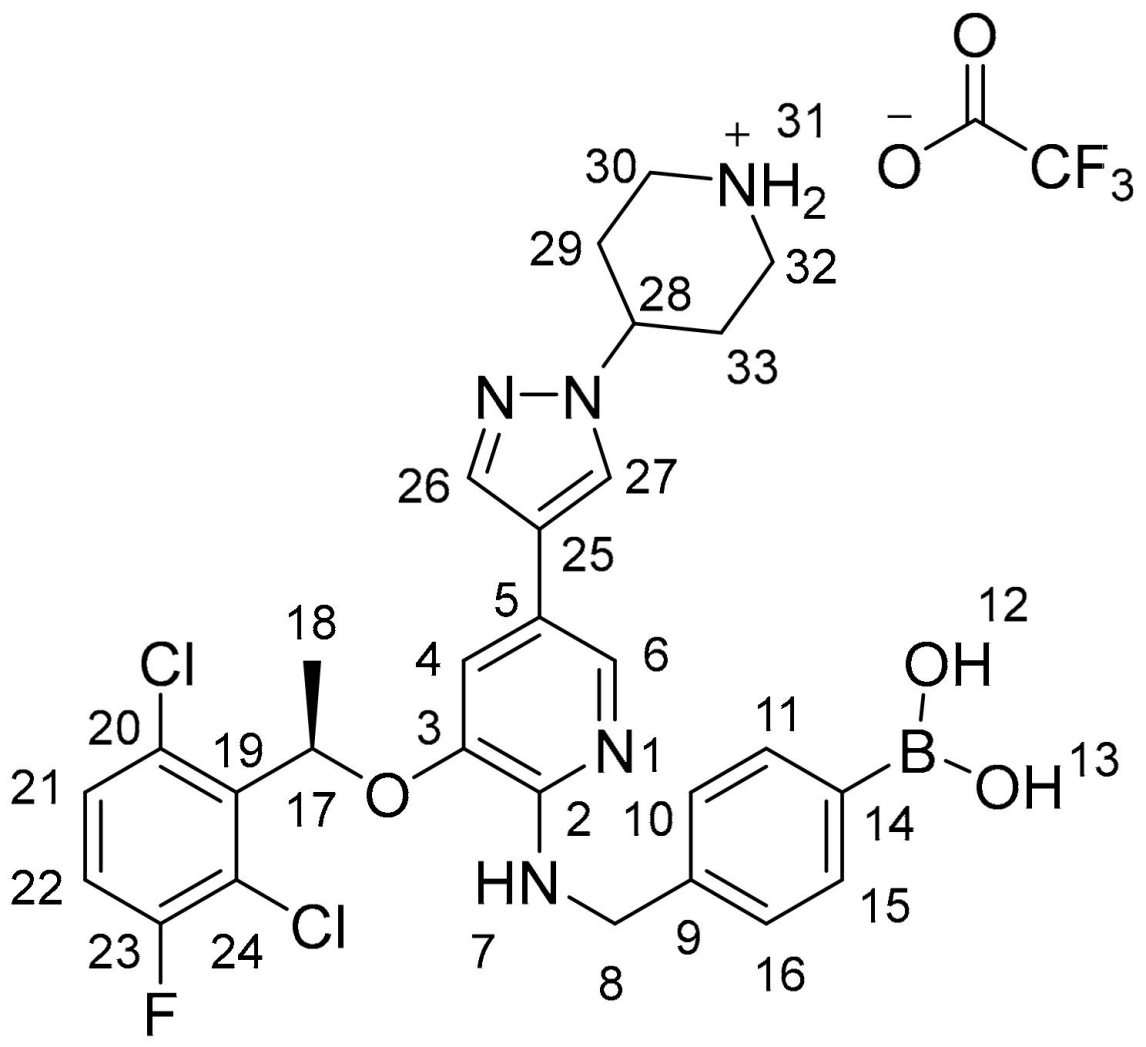
Structure and NMR numbering of prodrug **B**.

**Figure 9:**
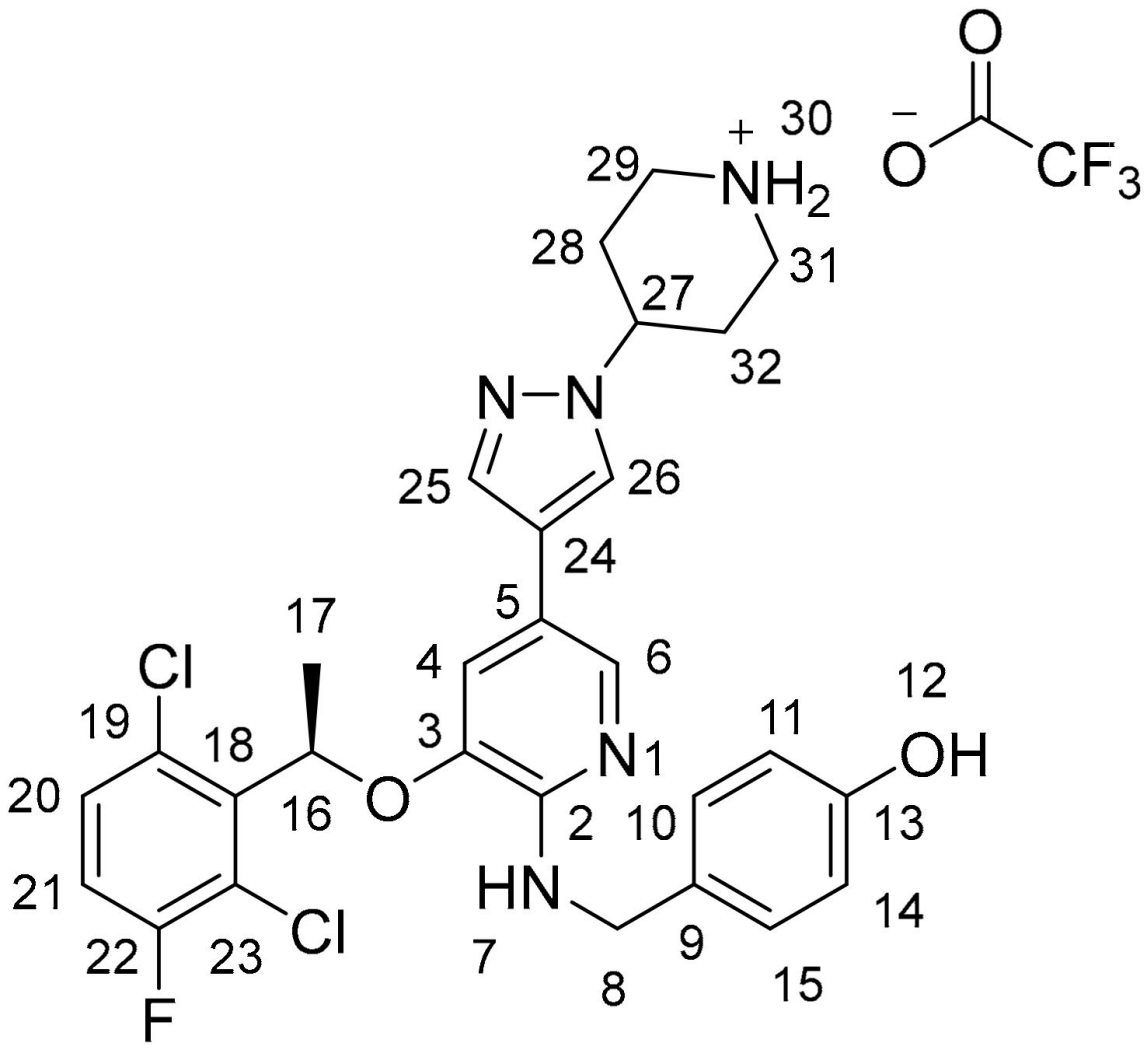
Structure and NMR numbering of compound **C**.

All solvents and reagents were obtained from commercial suppliers and, unless specified, used without further purification. Anhydrous DMF was bought from Sigma–Aldrich (Merck KGaA, MilliporeSigma, Darmstadt, Germany) over molecular sieves. Crizotinib was purchased from Triplebond Canada^®^ (TripleBond Corporation, Guelph Ontario, Canada). 4-(hydroxymethyl)-phenyl boronic acid pinacol ester **2** was bought from Boron Molecular^®^ (Boron Molecular Inc, Noble Park, Victoria, Australia). Aldehyde **4** was synthesized according to literature [29]. Preparative RP-HPLC purifications were performed on an Agilent 1200 Series system controlled by ChemStation^®^ software. The experimental conditions were as follows: stationary phase: ethylene bridged hybrid C18; column: XBridge BEH C18 OBD Prep Column, 130 Å, 10 μm, 19 mm × 250 mm (Waters Corp., MA, USA); flow rate: 17.06 mL/min, injection volume: 1–4 mL; column temperature: 25 °C; solvents: gradients of acetonitrile containing 0.1% trifluoroacetic acid (TFA) in Milli-Q water containing 0.1% trifluoroacetic acid. All HPLC-MS experiments were monitored on an Agilent 1260 Infinity (Agilent Technologies, Santa Clara, CA, USA) system using a Waters Atlantis T3 (Waters Corporation, Milford, MA, USA) column 150 mm × 4.6 mm coupled to a Bruker AmaZon SL ESI-IT mass spectrometer (Bruker Corporation, Billerica, MA, USA). Milli-Q water, containing 0.1% formic acid, and acetonitrile containing 0.1% formic acid, were used as eluents. A gradient of 1–99% acetonitrile over a period of 20 min was used. The Microanalytical Laboratory of the University of Vienna performed elemental analyses. The ESI-MS spectrometry was carried out with a Bruker Esquire3000 ion trap spectrometer (Bruker Daltonic, Bremen, Germany). Expected and experimental isotope distributions were compared. The ^1^H and ^13^C-NMR spectra were recorded in DMSO-*d*_6_ with a Bruker FT-NMR AV NEO 500 MHz spectrometer at 500.10 (^1^H) and 125.75 (^13^C) MHz at 298 K. The final products were characterized via 2D-NMR in DMSO-*d*_6_ with a Bruker FT-NMR AVIII 600 MHz spectrometer at 600.25 MHz (^1^H) and 150.93 MHz (^13^C). Chemical shifts (ppm) were referenced internal to the solvent residual peaks. For the description of the spin multiplicities the following abbreviations were used: s = singlet, d = duplet, t = triplet, q = quartet, m = multiplet. The NMR numbering schemes can be found in Figure 7, Figure 8 and Figure 9. The ^1^H and ^13^C-NMR spectra of prodrug **A**, prodrug **B** and compound **C** are depicted in Figures S9–S14.

Tert-butyl (*R*)-4-(4-(6-amino-5-(1-(2,6-dichloro-3-fluorophenyl)ethoxy)pyridin-3-yl)-1H-pyrazol-1-yl)piperidine-1-carboxylate (**1**).

Crizotinib (2.22 mmol, 1000 mg) was dissolved in 20 mL of tetrahydrofuran and di-tert-butyl dicarbonate (2.22 mmol, 484 mg) in 10 mL of tetrahydrofuran was added. The reaction was stirred at room temperature until no educt was indicated by TLC. The reaction mixture was taken up in ethyl acetate and washed with water and brine. The organic layers were combined, dried over MgSO_4_, and the solvent was removed under reduced pressure yielding a colorless oil. The oil was taken up in diethyl ether which was subsequently removed under reduced pressure giving a white powder. Yield: 1.16 g (96%). ^1^H-NMR (500.10 MHz, DMSO-*d_6_*) δ 7.98 (s, 1H), 7.76 (d, *J* = 1.7 Hz, 1H), 7.58 (dd, *J* = 9.0, 4.9 Hz, 1H), 7.54 (s, 1H), 7.45 (t, *J* = 9 Hz, 1H), 6.91 (d, *J* = 1.7 Hz, 1H), 6.10 (q, *J* = 6.6 Hz, 1H), 5.66 (s, 2H), 4.38–4.27 (m, 1H), 4.04 (bs, 2H), 2.92 (s, 2H), 2.01 (d, 2H), 1.81 (d, *J* = 6.6 Hz, 3H), 1.79– 1.70 (m, 2H), 1.43 (s, 9H) ppm. ESI-HRMS (*m*/*z*): calc: 550.1782, found: 550.1770 (M + H).

Tert-butyl (*R*)-4-(4-(5-(1-(2,6-dichloro-3-fluorophenyl)ethoxy)-6-((((4-(4,4,5,5-tetramethyl-1,3,2-dioxaborolan-2-yl)benzyl)oxy)carbonyl)amino)pyridin-3-yl)-1*H*-pyrazol-1-yl)piperidine-1-carboxylate (**3**).

Compound **1** (0.182 mmol, 100 mg), **2** (28 mg, 0.182 mmol), Cu(OAc)_2_ × H_2_O (0.546 mmol, 109 mg), potassium iodide (0.036 mmol, 6 mg), and Pd(OAc)_2_ (0.008 mmol, 2 mg) were dissolved in 1 mL of dimethyl sulfoxide and 4 mL of acetonitrile. The reaction vessel was set under an atmospheric pressure of carbon monoxide and shortly heated (5 min, 40 °C). The reaction mixture was stirred overnight and additional 50 mg of **1** was added. The reaction mixture was stirred for another 24 h, taken up in ethyl acetate and extracted with saturated (sat.) NaHCO_3_ and water. The combined aqueous solutions were extracted with ethyl acetate and the combined organic solutions were washed with brine, dried over MgSO_4_, and the solvent was removed under reduced pressure. The crude product was dried in vacuo overnight. HPLC investigations indicated a conversion of about 50%, and the crude product was used in the next step without further purification. ESI-MS (*m*/*z*): calc. 832.28, found: 832.28 (M + Na).

(*R*)-(4-((((3-(1-(2,6-Dichloro-3-fluorophenyl)ethoxy)-5-(1-(piperidin-4-yl)-1H-pyrazol-4-yl)pyridin-2-yl)carbamoyl)oxy)methyl)phenyl)boronic acid x 2TFA (**Prodrug A**).

Compound **3** was taken up in 2 mL of dichloromethane and 1 mL of trifluoroacetic acid was added. The resulting mixture was stirred for 2 h at room temperature and all volatiles were removed under reduced pressure. Purification of the final product was performed via preparative HPLC to yield a white powder (Figure 7). Yield: 21 mg (14% over 2 steps). ^1^H-NMR (600.25 MHz, DMSO-*d_6_*): δ 9.19 (s, 1H, N7), 8.54 (dd, *J* = 153.2, 10.0 Hz, 2H), 8.22 (d, *J* = 1.8 Hz, 1H, H6), 8.21 (s, 1H, H28), 8.06 (exch br s, 2H), 7.82 (s, 1H, H27), 7.79 (d, *J* = 8.0 Hz, 2H, H12/H16), 7.55 (dd, *J* = 9.0, 4.9 Hz, 1H, H22), 7.44 (t, *J* = 8.7 Hz, 1H, H23), 7.35 (d, *J* = 7.8 Hz, 3H, H11/H17/H4), 6.14 (q, *J* = 6.6 Hz, 1H, H18), 5.16–5.07 (m, 2H, H9), 4.54–4.48 (m, 1H, H29), 3.10 (dd, *J* = 22.8, 11.4 Hz, 2H, H31/H33), 2.22 (d, *J* = 12.0 Hz, 2H, H31/H33), 2.11 (td, *J* = 14.1, 3.4 Hz, 2H, H30/H34), 1.76 (d, *J* = 6.6 Hz, 3H, H30/H34) ppm. ^13^C-NMR (150.95 MHz, DMSO-*d*_6_) δ 157.00 (C24), 153.49 (C8), 146.82 (C3), 139.61 (C2), 138.66 (C10), 136.59 (C20), 136.46 (C6), 136.25 (C27), 134.15(C12/C16), 134.10 (C15*), 130.54 (C22), 128.70 (C21), 126.68 (C26), 126.44 (C11/C17), 125.92 (C28), 121.09 (C25), 117.89 (C5), 117.68 (C4), 117.54 (C23), 73.15 (C18), 65.57 (C9), 55.25 (C29), 42.23 (C31/C33), 28.68 (C30/C34), 18.56 (C19) ppm. ESI-HRMS (*m*/*z*): calc: 628.1698, found: 628.1701 (M + H). Anal. Calcd. for C_29_H_29_Cl_2_FN_5_O_5_ × 2CF_3_COOH (M_R_ = 855.15 g/mol): C, 43.28; H, 3.65; N, 8.19. Found: C, 43.05; H, 3.78; N, 8.47. * Only detected in 2D-NMR.

2-Picoline borane (**5**).

Compound **5** was prepared according to literature procedure with slight modifications [30]. Picoline (5 mmol, 466 mg), sodium bicarbonate (15 mmol, 1260 mg), and sodium borohydride (7.5 mmol, 280 mg) were dissolved in 1.9 mL of tetrahydrofuran. A solution of 15% water in tetrahydrofuran (1.9 mL) was added slowly while stirring vigorously. The product was filtered through celite and the solvent was removed under reduced pressure. The raw product was dissolved in ethyl acetate, extracted with citric acid (0.5 M) and brine. The organic solvent was dried with MgSO_4_ and removed in vacuo yielding white crystals. Yield: 437 mg (83%). ^1^H-NMR (500.10 MHz, DMSO-*d_6_*) δ = 8.68 (s, 1H), 7.77–7.74 (t, 1H), 7.32-7.30 (d, 1H), 7.23 (t, 1H), 7.26–7.22(t, 1H), 2.7 (s, 3H) ppm. ESI-MS (*m*/*z*): calc. 108.10, found: 108.10 (M + H).

Tert-butyl (*R*)-4-(4-(5-(1-(2,6-dichloro-3-fluorophenyl)ethoxy)-6-((4-(4,4,5,5-tetramethyl-1,3,2-dioxaborolan-2-yl)benzyl)amino)pyridin-3-yl)-1H-pyrazol-1-yl)piperidine-1-carboxylate (**6**).

Compound **1** (0.182 mmol, 100 mg) was dissolved in 3 mL of a mixture of acetic acid in methanol (1:10), **4** (0.182 mmol, 42 mg) and **5** (0.182 mmol, 20 mg) were added and the reaction mixture was stirred overnight. All volatiles were removed under reduced pressure and the crude product was used in the next step without further purification. ESI-MS (*m*/*z*): calc. 766.31, found: 766.45 (M + H).

(*R*)-(4-(((3-(1-(2,6-Dichloro-3-fluorophenyl)ethoxy)-5-(1-(piperidin-4-yl)-1H-pyrazol-4-yl)pyridin-2-yl)amino)methyl)phenyl)boronic acid x 2TFA (Prodrug **B**).

Compound **6** was taken up in 2 mL of dichloromethane and 1 mL of TFA was added. The reaction mixture was stirred for 30 min and all volatiles were removed under reduced pressure. Purification of the final product was performed via preparative HPLC to yield a white powder (Figure 8). Yield: 60 mg (38% over 2 steps)). ^1^H-NMR (500.10 MHz, DMSO-*d_6_*): δ 8.53 (exch br d, *J* = 124.4 Hz, 2H, H31), 7.95 (exch br s, 2H, H12/H13), 7.92 (s, 1H, H26/H27), 7.74 (s, 1H, H6), 7.71 (d, *J* = 7.9 Hz, 2H, H11 + H15), 7.61–7.55 (m, 2H, H26/H27 + H21), 7.47 (t, *J* = 8.7 Hz, 1H, H22), 7.26 (d, *J* = 8.0 Hz, 2H, H10 + H16), 6.90 (s, 1H, H4), 6.17 (q, *J* = 6.5 Hz, 1H, H17), 4.62 (dd, *J* = 46.0, 11.5 Hz, 2H, H8), 4.46 (tt, *J* = 10.8, 3.9 Hz, 1H, H28), 3.40 (d, *J* = 13.1 Hz, 2H, H30/H32), 3.07 (dd, *J* = 23.4, 11.5 Hz, 2H, H30/H32), 2.17 (d, *J* = 12.9 Hz, 2H, H29/H33), 2.07 (dd, *J* = 23.3, 11.9 Hz, 2H, H29/H33), 1.84 (d, *J* = 6.6 Hz, 3H, H18) ppm. ^13^C-NMR (150.95 MHz, DMSO-*d*_6_) δ 156.88 (C23), 147.90 (C2*), 142.50 (C9*), 139.54 (C3), 136.38 (C19), 135.34 (C26/C27), 134.09 (C11/C15), 132.53 (C14*), 130.71 (C21), 128.77 (C20), 125.96 (C10/C16), 124.36 (C26/C27), 121.07 (C24), 118.65 (C5), 117.68 (C22), 116.37 (C25), 114.25 (C4), 72.41 (C17), 55.13 (C28), 43.84 (C8), 42.28 (C30/C32), 28.74 (C29/C33), 18.70 (C18) ppm. ESI-HRMS (*m*/*z*): calc: 584.1802, found: 584.1789. Anal. Calcd. for C_28_H_29_BCl_2_FN_5_O_3_ × 2CF_3_COOH (M_R_ = 888.67 g/mol): C, 45.61; H, 3.66; N, 8.06. Found: C, 45.93; H, 4.05; N, 8.34. * Only detected in 2D-NMR.

Tert-butyl (R)-4-(4-(5-(1-(2,6-dichloro-3-fluorophenyl)ethoxy)-6-((4-hydroxybenzyl)amino)pyridin-3-yl)-1H-pyrazol-1-yl)piperidine-1-carboxylate (**7**).

**1** (0.182 mmol, 100 mg) was dissolved in 3 mL of a mixture of acetic acid in methanol (1:10). 4-Hydroxybenzaldehyde (0.182 mmol, 22 mg) and **5** (0.182 mmol, 20 mg) were added and the reaction mixture was stirred overnight. Next day, additional 4-hydroxybenzaldehyde (0.091 mmol, 10 mg) and **5** (0.182 mmol, 20 mg) were added and the reaction mixture was stirred for 3 h. All volatiles were removed and the product was purified using silica column chromatography (EtOAc/PE 1:1) yielding a white powder. Yield: 59 mg (50%). ^1^H-NMR (500.10 MHz, DMSO-*d_6_*) δ 9.22 (s, 1H), 7.93 (s, 1H), 7.78 (s, 1H), 7.56 (dd, *J* = 8.8, 4.9 Hz, 1H), 7.52 (s, 1H), 7.44 (t, *J* = 8.8 Hz, 1H), 7.11 (d, *J* = 8.3 Hz, 2H), 6.85 (s, 1H), 6.66 (d, *J* = 8.3 Hz, 2H), 6.18 (t, *J* = 6.3 Hz, 1H), 6.10 (t, *J* = 6.6 Hz, 1H), 4.44 (ddd, *J* = 36.0, 14.6, 6.0 Hz, 2H), 4.36–4.27 (m, 1H), 4.02 (s, 2H), 2.90 (s, 2H), 1.98 (d, *J* = 9.2 Hz, 3H), 1.80 (d, *J* = 6.6 Hz, 3H), 1.78–1.69 (m, 2H), 1.41 (s, 9H). ESI-MS (*m*/*z*): calc. 656.22, found: 656.42 (M + H).

(*R*)-4-(((3-(1-(2,6-Dichloro-3-fluorophenyl)ethoxy)-5-(1-(piperidin-4-yl)-1H-pyrazol-4-yl)pyridin-2-yl)amino)methyl)phenol (Compound **C**).

Compound **7** (0.091 mmol, 59 mg) was dissolved in 2 mL of dichloromethane and 1 mL of trifluoroacetic acid was added. The reaction mixture was stirred for 30 min and all volatiles were removed. Purification of the final product was performed via preparative HPLC to yield a yellow powder (Figure 9). Yield: 36 mg (44%). ^1^H-NMR (500.10 MHz, DMSO-*d_6_*) δ 9.34 (exch br s, 1H, H12), 8.60 (d, *J* = 121.9 Hz, 2H, H30), 8.00 (s, 1H, H25/H26), 7.72 (s, 1H, H6), 7.65 (s, 1H, H25/H26), 7.59 (dd, *J* = 9.0, 4.9 Hz, 1H, H20), 7.47 (t, *J* = 8.7 Hz, 1H, H21), 7.15 (d, *J* = 8.5 Hz, 2H, H10/H15), 6.98 (s, 1H, H4), 6.71 (d, *J* = 8.5 Hz, 2H, H11/H14), 6.22 (q, *J* = 6.5 Hz, 1H, H16), 4.60–4.45 (m, 4H, H8/H27), 3.41 (d, *J* = 12.8 Hz, 2H, H29/H31), 3.09 (q, *J* = 11.5 Hz, 2H, H29/H31), 2.19 (d, *J* = 12.2 Hz, 2H, H28/H32), 2.08 (m, 2H, H28/H32), 1.85 (d, *J* = 6.6 Hz, 3H, H17). ^13^C-NMR (150.95 MHz, DMSzO-*d*_6_) δ 157.35 (C22), 156.94 (C13), 146.55 (C2*), 140.75 (C3), 135.98 (C25/C26), 135.93 (C5*), 131.25 (C20), 129.16 (C19), 128.87 (C10/C15), 125.33 (C25/C26), 121.49 (C23), 118.28 (C21), 116.43 (C24*), 115.74 (C4), 115.55 (C11/14), 115.35 (C9), 73.44 (C16), 55.67 (C27), 44.13 (C8), 42.96 (C29/C31) 29.16 (C28/C32), 19.85 (C17) ppm. ESI-HRMS (*m*/*z*): calc: 556.1677, found: 556.1683 (M + H). Anal. Calcd. for C_28_H_29_BCl_2_FN_5_O_2_ × 3CF_3_COOH × H_2_O (M_R_ = 916.54 g/mol): C, 44.55; H, 3.63; N, 7.64. Found: C, 44.50; H, 3.28; N, 7.55. * Only detected in 2D-NMR.

### 4.2. Docking

Prodrug structures were drawn using the ChemBioDraw^®^ Office Suite (PerkinElmer, Waltham, MA, USA) and conformation energy was minimized using ChemBio3D^®^ (PerkinElmer, Waltham, MA, USA). This structure was further optimized via DFT calculations implemented in the Gaussian09 program package (Gaussian Inc, Wallingford, CT, USA) [31], using the B3LYP functional and the 6-31G(d) basis set. The Protein Data Bank files PDB ID: 2XP2 (ALK) and 2WGJ (c-MET) were used as models in complex with crizotinib. Addition and merging of non-polar hydrogens of the receptors and the assignment of rotatable bonds of the prodrugs were performed using the AutoDockTools 1.5.6 software (Molecular Graphics Laboratory, La Jolla, CA, USA) [32]. The rotatable bonds of the used protein models were considered as being rigid. The grid size for ALK was set to 40 × 40 × 40 points (grid spacing: 1.000 Å) and a grid center of 30.292, 46.787 and 8.649 (center of mass of crizotinib in 2XP2). Additionally, the grid size of 2WGJ was set to 40 × 40 × 40 with a grid center of 21.316, 83.731, and 4.561 (center of mass of crizotinib in 2WGJ). Visualization of grid boxes are shown in Figure S1. Docking studies were performed using AutoDock Vina [33]. Validation of the docking process by AutoDock Vina was performed by extracting and redocking the ligands as they were in the original PDB files and RMS values were compared using PyMol (<2 Å). All docking procedures were performed in quintuplicates. As boron atoms are not included in the internal force field of AutoDock Vina, an aromatic carbon was used for mimicking as the closest atom type available in the force field. Results of the docking experiments are summarized in Table S1. All 3D models were visualized using PyMol [34] and 2D interactions using the LigPlot^+^ software package (Figure S2; hydrogen bond calculation parameters: maximum H-A distance 3.5; maximum D-A distance 3.5) [35].

### 4.3. In silico-ADME Prediction

All calculations were performed on the SwissADME web page [26] using crizotinib and both prodrugs with a protonated piperidine moiety.

### 4.4. Kinase Screening

The ALK and c-MET kinase inhibitory potential of both prodrugs was evaluated using the SelectScreen^®^ Biochemical Kinase Profiling Service at Life Technologies (Thermo Fisher Scientific, Madison, WI, USA). The test compounds were screened in a final concentration of 1% DMSO using the Z′-LYTE^®^ Assay.

### 4.5. H_2_O_2_ Activation Assay

10 mM stock solutions of the drugs were prepared in DMSO and diluted 1:10 with PB7.4. Additionally, a H_2_O_2_ solution (1 mM) in PB7.4 was prepared. The activation was started by addition of 5 µL of drug solution and 25 µL of H_2_O_2_ to 70 µL of PB7.4 and incubated at 37 °C for 1 h. At time point 0 and every 8 min, the sample were measured via HPLC. All measurements were performed on a ThermoFisher Ultimate 3000RS system using a Water’s Acquity BEH C18 column (3 × 50 mm, 1.7 µm) and a 5%–95% gradient of acetonitrile (+0.1% TFA) in water (+0.1% TFA). Degradation of the prodrugs and formation of crizotinib were detected at 230 nm. The results are visualized in Figure S6 for prodrug **A** and in Figure S7 for prodrug **B**.

### 4.6. Stability Assay

The 10 mM stock solutions of the drugs were prepared in DMSO and diluted 1:10 with PB7.4. 1 µL of those solutions were added to 19 µL of PB7.4, R10 medium (RPMI-1640 medium containing 10% of fetal calf serum) or pure fetal calf serum. All samples were incubated at 37 °C for up to 72 h. At different time points, macromolecules were precipitated by addition of 40 µL of acetonitrile, vortexed and centrifuged for 10 min at 6000 rpm. The supernatant was extracted and investigated via HPLC-MS measurements. Peaks were evaluated at 260 nm and their relative areas under the curve (AUC) were visualized in Figure 4.

### 4.7. Cell Culture

The H1993 (ATCC^®^ CRL-5909^TM^), H2228 (ATCC^®^ CRL-5935^TM^) and the human primary lung fibroblasts HLF (ATCC^®^ PCS-201-013^TM^) were obtained from the American Type Culture Collection (ATCC^®^). The renal cell carcinoma cell line RUMH was established at the Institute of Cancer Research, Vienna, Austria. Cell lines were kept under standard cell culture conditions in RPMI-1640 growth medium supplemented with 10% fetal bovine serum (PAA, Linz, Austria) in 5% CO_2_-containing humidified atmosphere at 37 °C.

### 4.8. Cytotoxicity Assay

All samples were dissolved in DMSO and diluted to the indicated concentrations (final concentrations of DMSO were below 1%). Preincubation of the (pro-)drugs with H_2_O_2_ was performed in aqueous 0.9% sodium chloride solution with 1.6 equivalents of H_2_O_2_ at room temperature for 2 h. Depending on the proliferation rate, cells were seeded at a density of 3.5–8 × 10^3^ cells/well in 96-well plates (100 µL/well). After 24 h, cells were exposed to drugs at the indicated concentrations for 72 h. Viability was measured using 3-(4,5-dimethylthiazol-2-yl)-2,5-diphenyltetrazolium bromide (MTT) based vitality assay (EZ4U; Biomedica, Vienna, Austria) following the manufacturer’s recommendations. The IC_50_ values (drug concentrations causing 50% reduction in viable cell number in comparison to untreated control) were calculated using GraphPad Prism software (point-to-point function from a full dose-response curve; GraphPad Software Inc, San Diego, CA, USA).

### 4.9. ROS Assay by Flow Cytometry

To detect production of ROS, 5 × 10^5^ cells were incubated with 10 µM DCFH-DA (2′,7′-dichlorofluorescein diacetate, Sigma-Aldrich) in Hank’s Balanced Salt Solution (HBSS) for 1 h at 37 °C. Subsequently, mean fluorescence signal of 30,000 single cells per sample was measured by flow cytometry using a BD LSR Fortessa^TM^ instrument (BD Biosciences, Franklin Lakes, NJ, USA). Data were analyzed by FlowJo (BD Biosciences, San Jose, CA, USA) and GraphPad Prism software (GraphPad Software Inc, San Diego, CA, USA).

## Figures and Tables

**Table 1:** In silico adsorption, distribution, metabolization, excretion (ADME) calculations to predict the “drug-likeness” of prodrug **A**, prodrug **B** and crizotinib using SwissADME.

Models	Prodrug A	Prodrug B	Crizotinib
Molecular weight	629.29	585.28	451.34
Num. H-bond acceptors	8	6	4
Num. H-bond donors	4	4	2
TPSA (≤140 Å)	135.34 Å^2^	109.04 Å^2^	82.57 Å^2^
Consensus Log Po/w	3.23	3.09	3.73
Log S (ESOL)	−6.15	−6.29	−5.05
GI absorption	Low	High	High
Blood–brain-barrier (BBB)-permeant	No	No	No
CYP1A2 inhibitor	No	No	Yes
CYP2C9 inhibitor	No	No	No
CYP2C19 inhibitor	Yes	Yes	No
CYP2D6 inhibitor	No	No	No
CYP3A4 inhibitor	Yes	Yes	No
P-glycoprotein substrate	No	Yes	Yes
Log Kp (skin permeation)	−7.05 cm/s	−6.50 cm/s	−6.43 cm/s
PAINS	0 alert	0 alert	0 alert
Bioavailability score	0.55	0.55	0.55
Lipinski requirements	Yes; 1 violation *	Yes; 1 violation *	Yes; 0 violation

TPSA: topological polar surface area; Log S (ESOL): aqueous solubility; CYP: cytochrome P450; PAINS: pan-assay interference compounds. * Molecular weight > 500 g/mol.

**Table 2:** Cytotoxicity of prodrug **A**, prodrug **B,** compound **C**, and crizotinib was determined by MTT assay after 72 h treatment. The three cancer cell lines (i.e., H1993, H2228, RUMH) and non-malignant human lung fibroblasts (HLF) cells were investigated. Ratios were calculated for ±H_2_O_2_.

Drugs	IC_50_ (µM)						
	H1993	Ratio	H2228	Ratio	RUMH	Ratio	HLF
Prodrug A	9.73 ± 2.63	1.22	23.14 ± 0.47	1.62	13.97 ± 3.92	1.57	16.79 ± 1.38
A + H_2_O_2_	7.96 ± 1.38		14.31 ± 1.73		8.88 ± 1.54		
Prodrug B	10.21 ± 1.68	0.87	8.3 ± 0.42	0.92	5.05 ± 0.44	0.91	8.99 ± 0.36
B + H_2_O_2_	11.79 ± 1.44		9.03 ± 0.81		5.58 ± 1.51		
Crizotinib	3.18 ± 0.26	1.12	13.73 ± 0.33	1.04	6.08 ± 1.51	1.00	6.32 ± 0.39
Crizotinib + H_2_O_2_	2.84 ± 0.23		13.21 ± 0.32		6.06 ± 1.7		
Compound C	9.96 ± 2.69		7.23 ± 0.28		5.94 ± 1.09		4.03 ± 0.04

## Supplementary Materials

The following are available online: Figure S1: grid sizes of the docking studies, Figure S2: 2D interactions of the prodrugs with the kinases. Table S1: docking scores. Figure S3: BOILED-Egg plots. Figure S4: Stability assay. Table S2 and Figure S5: kinase inhibition data. Figures S6 and S7: H_2_O_2_ prodrug activation data. Figure S8: Docking studies of compound **C**. Table S3: Docking scores of compound **C**. Figures S9–S14: ^1^H and ^13^C NMR of prodrugs **A** and **B** and compound **C**.
