# Supplementary material for: Reactive Oxygen Species (ROS)-Sensitive Prodrugs of the Tyrosine Kinase Inhibitor Crizotinib
Source: Molecules. 2020 Mar 4;25(5):1149. doi: 10.3390/molecules25051149 (PMC7179202; doi:10.3390/molecules25051149)
Supplement: Supplementary file 1 [file molecules-25-01149-s001.pdf]

Supplementary information

Reactive oxygen species (ROS)-sensitive prodrugs of the tyrosine kinase inhibitor crizotinib

Bjoern Bielec[1], Isabella Poetsch[1,2], Esra Ahmed[1], Petra Heffeter[2,3], Bernhard K. Keppler[1,3], Christian R. Kowol[1,3*]

[1] Institute of Inorganic Chemistry, Faculty of Chemistry, University of Vienna, Waehringer Strasse 42, 1090 Vienna, Austria. E-Mail: christian.kowol@univie.ac.at; Fax: +43-1-4277-52680; Tel: +43-1-4277-52609.
[2] Institute of Cancer Research, Medical University of Vienna, Borschkegasse 8a, 1090 Vienna, Austria
[3] Research Cluster ''Translational Cancer Therapy Research'', University of Vienna, Waehringer Strasse 42, 1090 Vienna, Austria
* Correspondence: christian.kowol@univie.ac.at; Tel.: +43-1-4277-52680

Docking

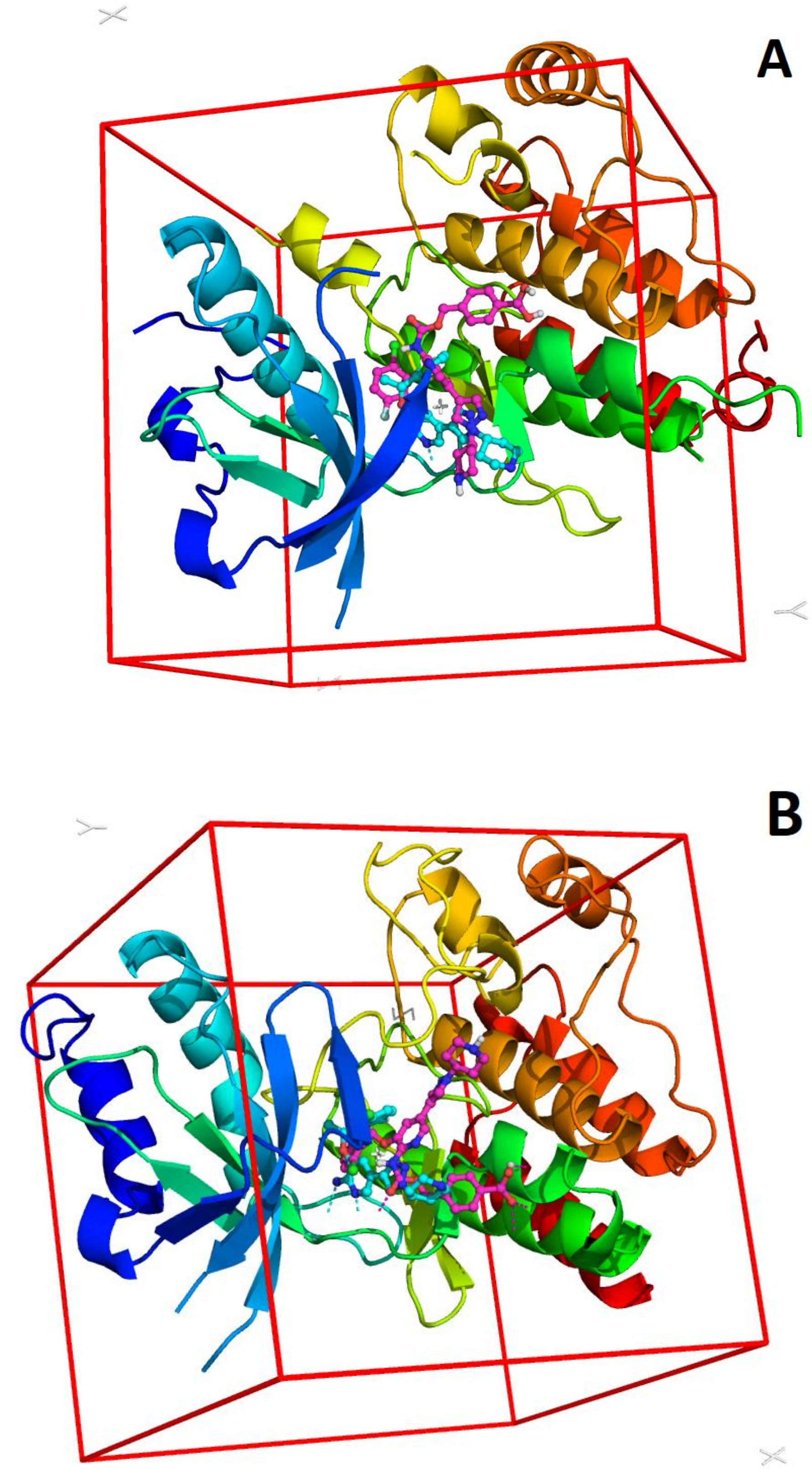

*Figure S1. (A) Grid sizes for ALK (PDB ID: 2XP2) in complex with crizotinib and prodrug **A.** (B) Grid sizes for c-MET (PDB ID: 2WGJ) in complex with crizotinib and prodrug **B**. Proteins are depicted in ribbon representation while compounds are shown in capped stick representation. Pictures were generated using PyMol.*

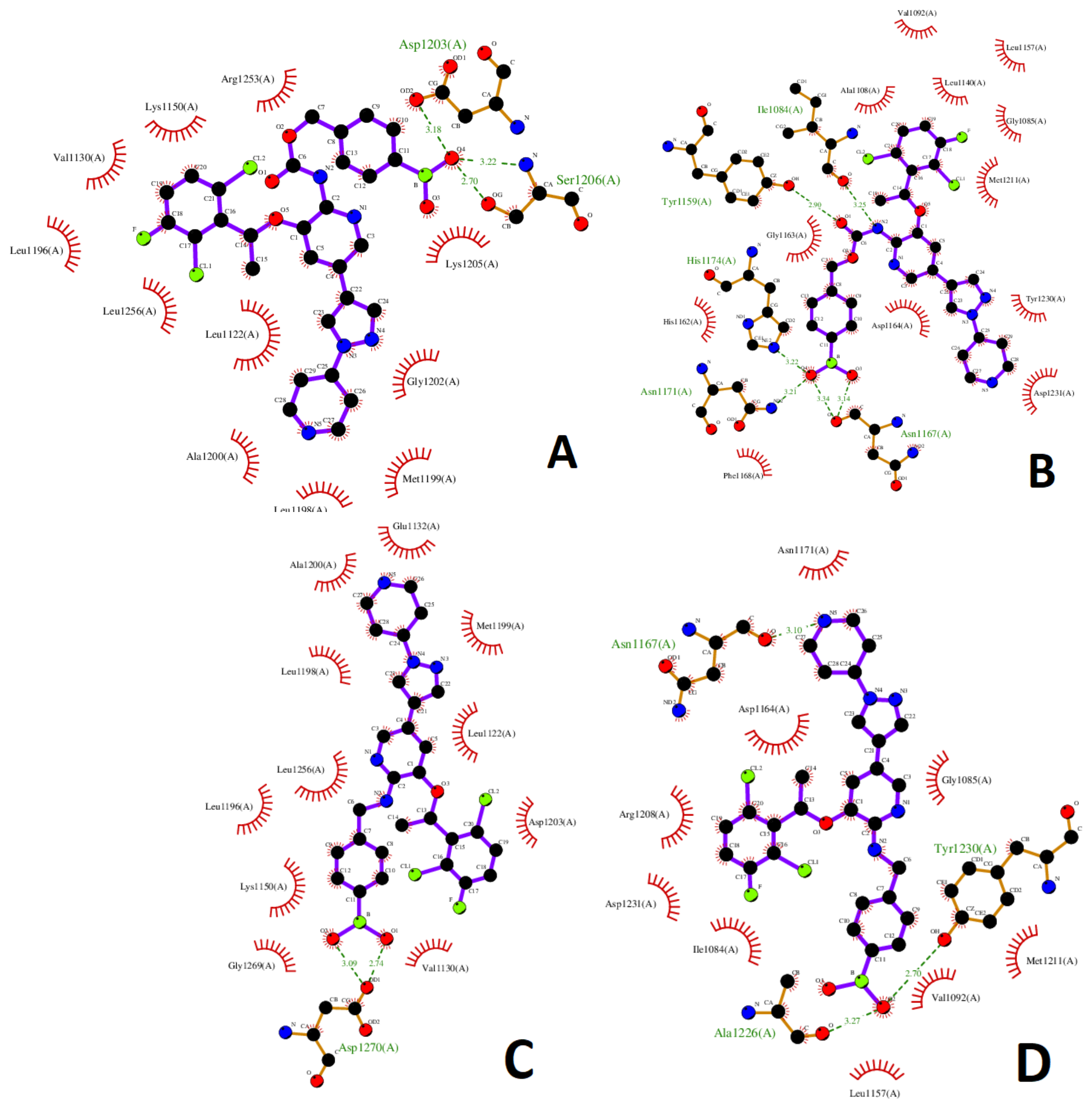

*Figure S2. 2D interactions of prodrug **A** with ALK (A) and c-MET (B) and prodrug **B** with ALK (C) and c-MET (D) as calculated by AutoDock Vina plotted with the LigPlot+ software package. Hydrogen bonds and bond lengths are visualized in green dashed lines, while an arc represents hydrophobic contacts with spokes radiating towards the ligand atoms they contact. The contacted atoms are shown with spokes radiating back.*

Table S1. Results of the docking experiments for ALK and c-MET with redocked crizotinib and both prodrugs.

ALK		c-MET	
Drug	Docking Score [kcal/mol]	Drug	Docking Score [kcal/mol]
Crizotinib	- 8.9	Crizotinib	- 10.4
Prodrug **A**	- 8.8	Prodrug **A**	- 9.7
Prodrug **B**	- 9.3	Prodrug **B**	- 9.7

ADME prediction

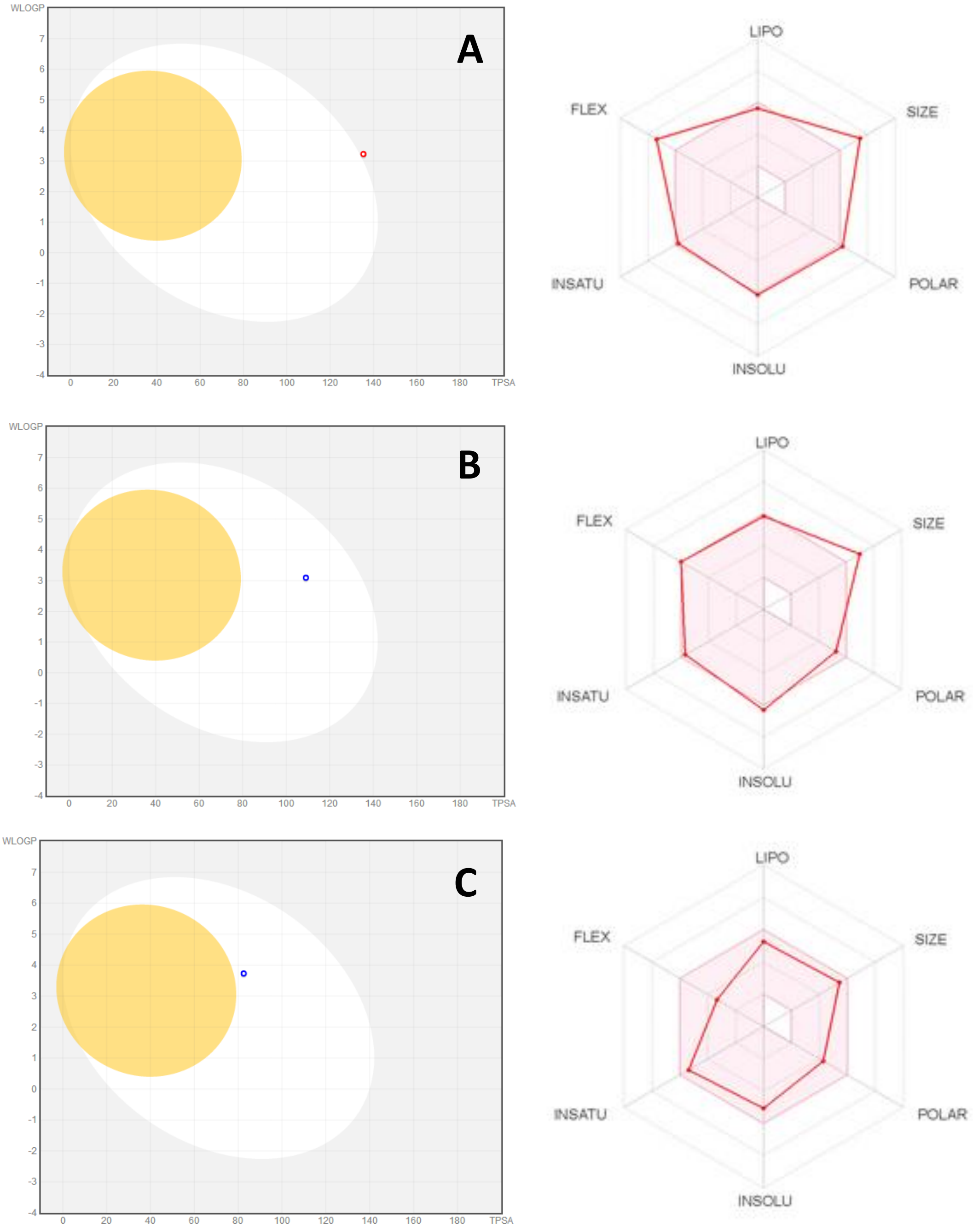

Figure S3. Boiled-egg plots of prodrug ***A*** *(A), prodrug* ***B*** *(B) and crizotinib (C). Drugs marked as blue represents the drug as P-glycoprotein substrate, red as non-P-glycoprotein substrate.*

Stability assay

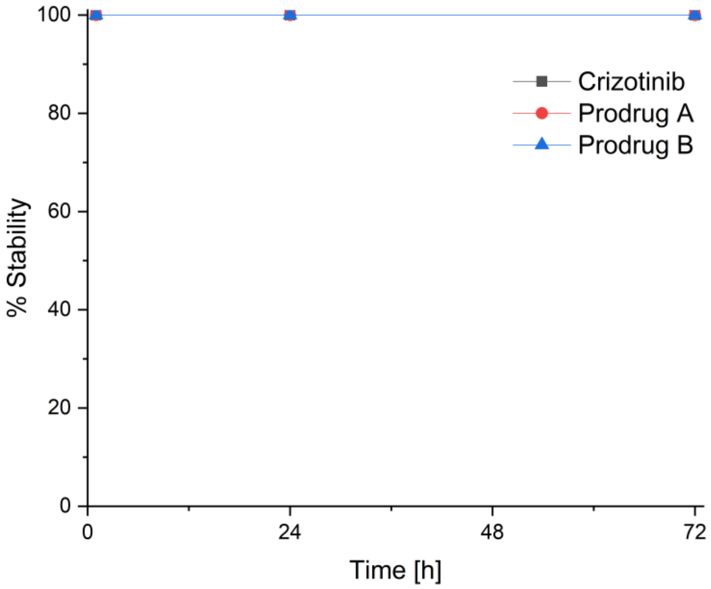

Figure S4. Stability data for crizotinib (black), prodrug A (red) and prodrug B (blue) in phosphate buffer at pH 7.4 for 72 h.

Kinase Inhibition assay

Table S2. Results of the cell-free kinase screening experiments.

	IC_{50} ALK inhibition (nM)	Prodrug factor*	IC_{50} c-Met inhibition (nM)	Prodrug factor*
Crizotinib	21	-	5	-
Prodrug **A**	472	22	55	11
Prodrug **B**	333	16	684	137

$$* \textbf{ Prodrug Factor} = \frac{\textbf{IC50 (Prodrug)}}{\textbf{IC50 (Crizotinib)}}$$

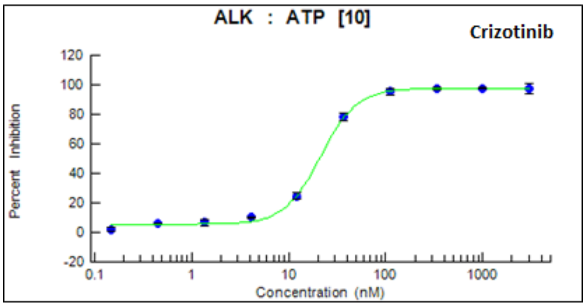

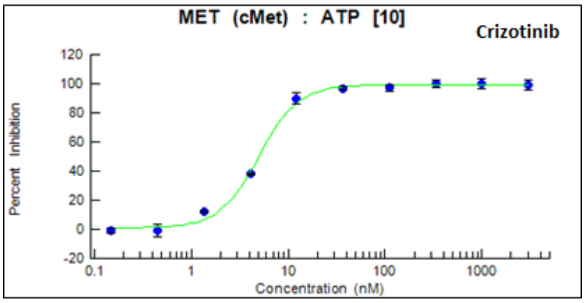

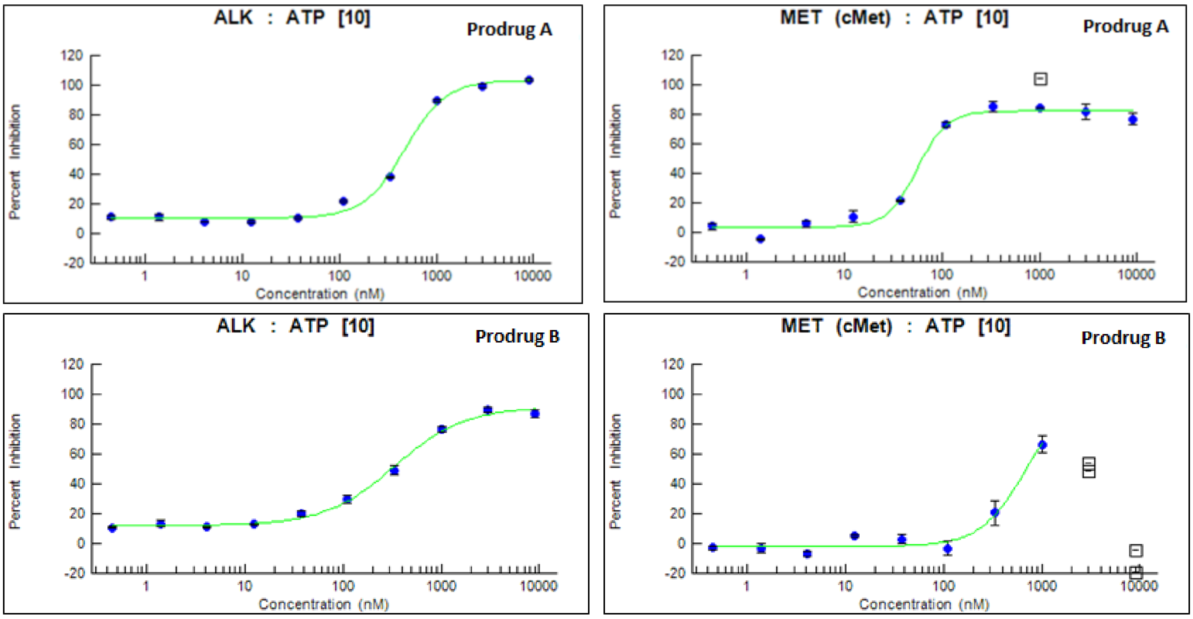

Figure S5. Data points of the cell-free kinase inhibition assay (in case of prodrug B and c-MET the two highest data points did not pass the criteria of the "development reaction interference" and are consequently excluded).

Activation

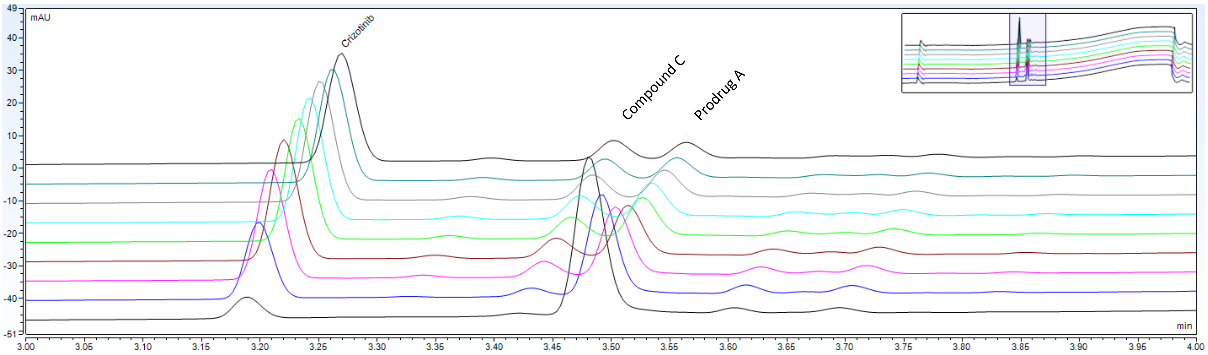

*Figure S6. Release of crizotinib from prodrug **A** after incubation with hydrogen peroxide over 1 h.*

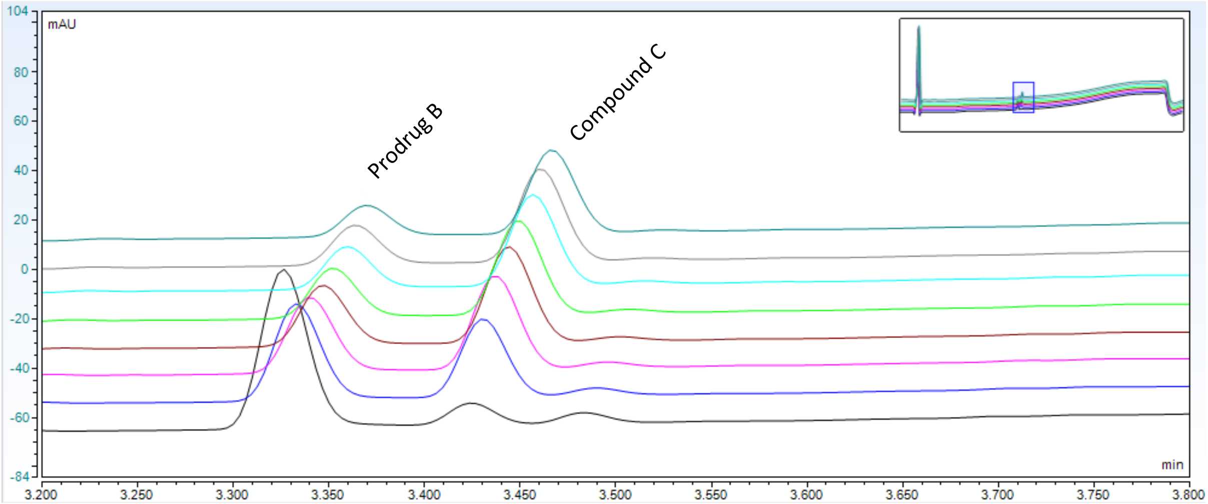

*Figure S7. Release of compound **C** from prodrug **B** after incubation with hydrogen peroxide over 1 h.*

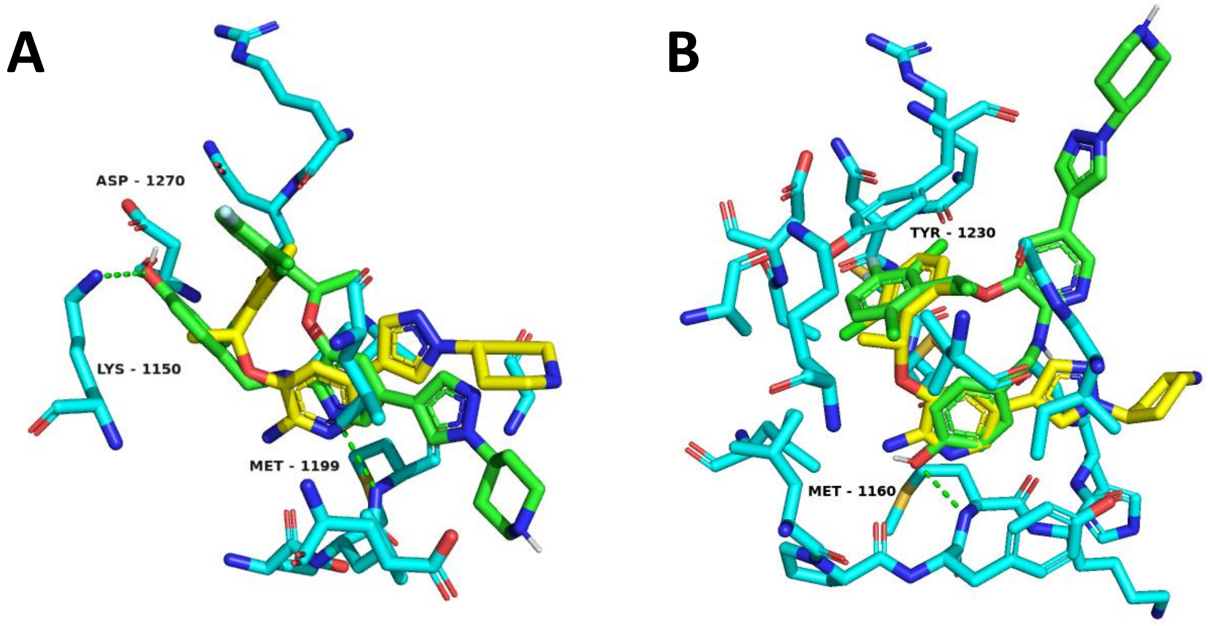

*Figure S8. A) Spatial interaction of compound **C** in complex with ALK (PDB ID: 2XP2). Green lines display hydrogen bonds between the pyridinyl nitrogen and Met-1199 and the phenol group and Lys-1150. B) Spatial interaction of compound **C** and c-MET (PDB ID: 2WGJ). ALK is shown in cyan, crizotinib is colored in yellow and compound **C** is shown in green.*

*Table S3. Lowest docking score of crizotinib and compound **C** when docked to ALK and c-MET.*

ALK		c-MET	
Drug	Docking Score [kcal/mol]	Drug	Docking Score [kcal/mol]
Crizotinib	- 8.9	Crizotinib	- 10.4
Compound **C**	- 8.6	Compound **C**	- 9.7

NMR spectra

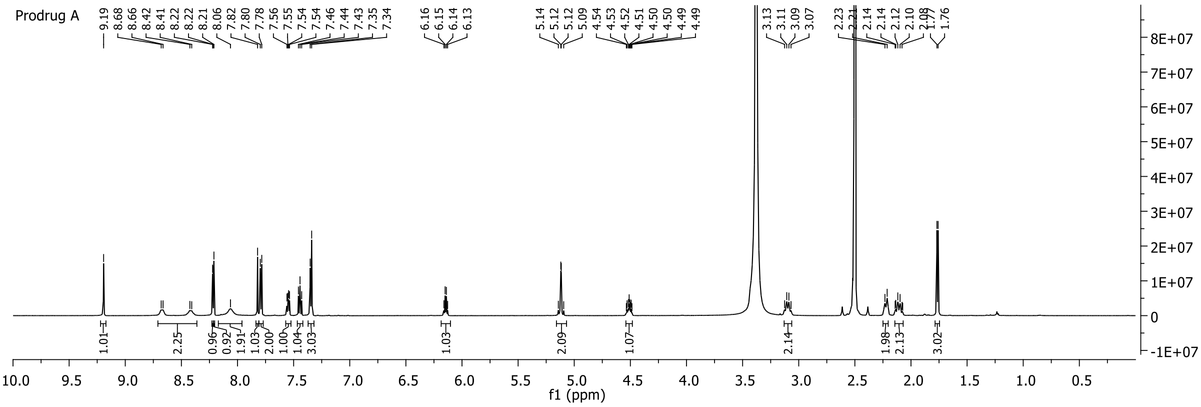

*Figure S9. 1H NMR of prodrug **A***

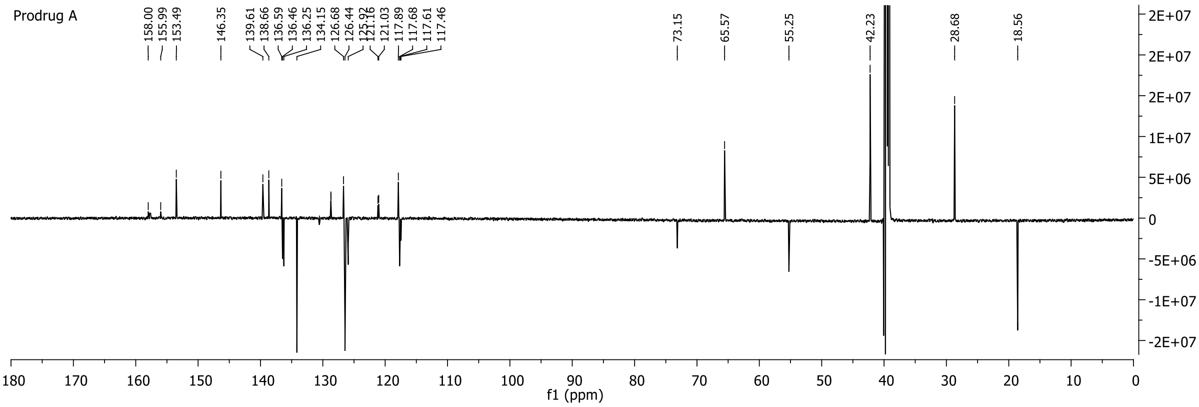

*Figure S10. 13C NMR of prodrug **A***

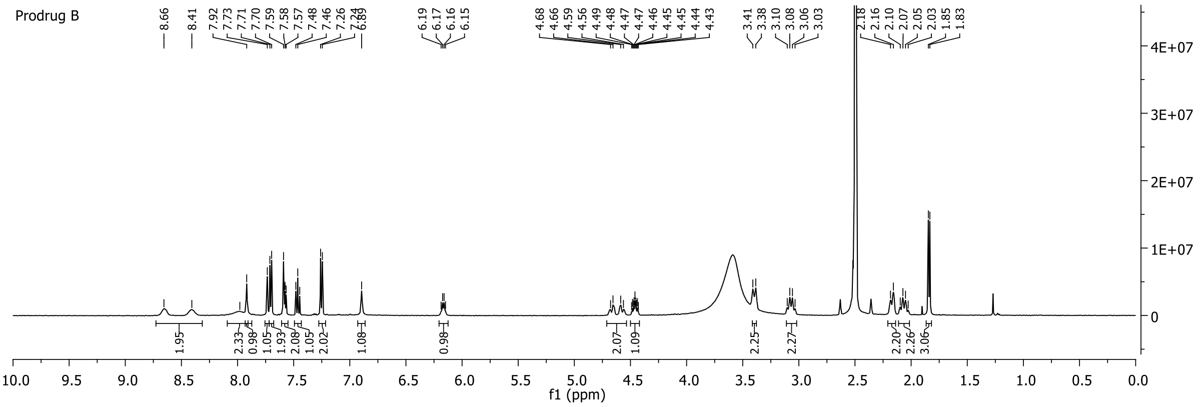

*Figure S11. 1H NMR of prodrug **B***

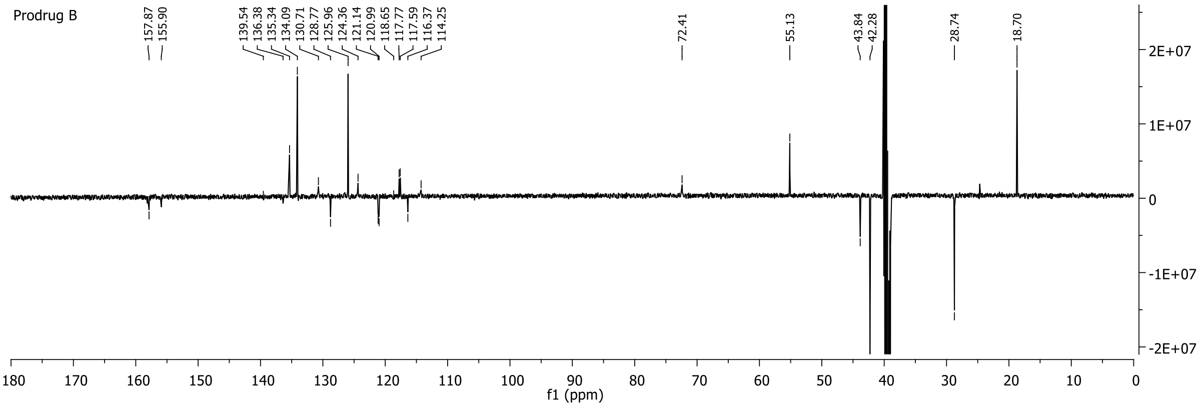

Figure S12. 13C NMR of prodrug ***B***

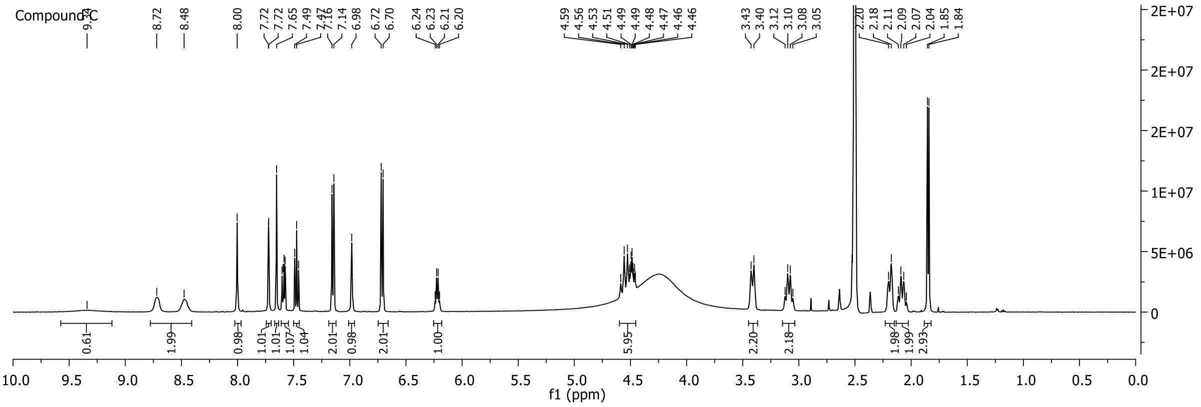

Figure S13. 1H NMR of compound ***C***

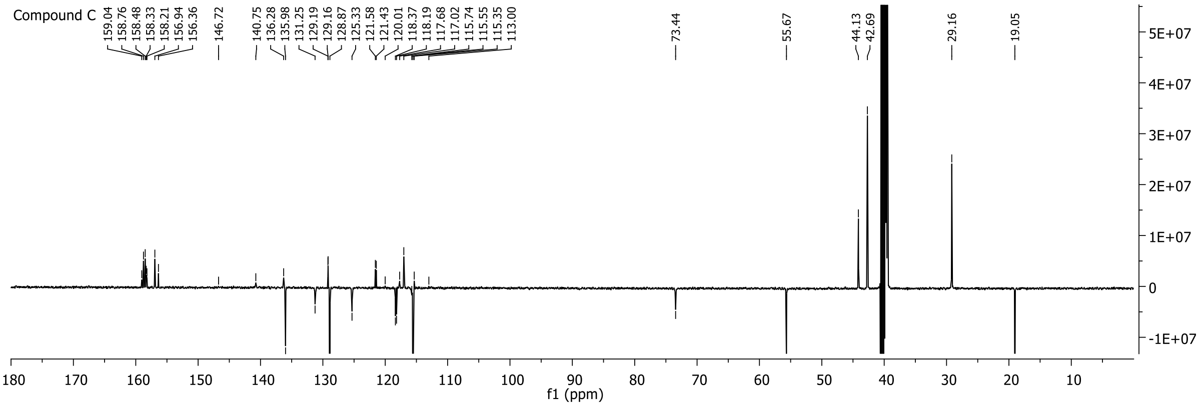

Figure S14. 13C NMR of compound ***C***
